# Global solutions of aggregation equations and other flows with random diffusion

**DOI:** 10.1007/s00440-022-01171-8

**Published:** 2022-10-31

**Authors:** Matthew Rosenzweig, Gigliola Staffilani

**Affiliations:** Massachusetts Institute of Technology, Cambridge, USA

**Keywords:** 35Q35, 35Q49, 35Q70, 35R60, 60H50

## Abstract

Aggregation equations, such as the parabolic-elliptic Patlak–Keller–Segel model, are known to have an optimal threshold for global existence versus finite-time blow-up. In particular, if the diffusion is absent, then all smooth solutions with finite second moment can exist only locally in time. Nevertheless, one can ask whether global existence can be restored by adding a suitable noise to the equation, so that the dynamics are now stochastic. Inspired by the work of Buckmaster et al. (Int Math Res Not IMRN 23:9370–9385, 2020) showing that, with high probability, the inviscid SQG equation with random diffusion has global classical solutions, we investigate whether suitable random diffusion can restore global existence for a large class of active scalar equations in arbitrary dimension with possibly singular velocity fields. This class includes Hamiltonian flows, such as the SQG equation and its generalizations, and gradient flows, such as those arising in aggregation models. For this class, we show global existence of solutions in Gevrey-type Fourier–Lebesgue spaces with quantifiable high probability.

## Introduction

### Motivation

To motivate the problem addressed in this article, let us consider the two-dimensional aggregation-diffusion equation

1.1 $$\begin{aligned} {\left\{ \begin{array}{ll} {\partial }_t\theta = {{\,\mathrm{\textrm{div}}\,}}(\theta \nabla {\textsf{g}}*\theta ) + \nu \Delta \theta \\ \theta |_{t=0} = \theta ^0 \end{array}\right. } \qquad (t,x) \in {\mathbb {R}}_+\times {\mathbb {R}}^2. \end{aligned}$$

Here, ${\textsf{g}}(x) = \frac{1}{2\pi }\ln |x|$ is the Newtonian potential on ${\mathbb {R}}^2$ and $\nu \ge 0$ is the diffusion strength. It is natural to assume that $\theta ^0\ge 0$ and consider solutions $\theta \ge 0$, as $\theta$ is supposed to represent a density. If $\nu >0$, then Eq. (1.1) is known as the parabolic-elliptic Patlak–Keller–Segel (PKS) equation, which is a model for the aggregation of cells by chemotaxis [68, 79, 82]. If $\nu =0$, then the equation is the gradient flow of the Newtonian energy with respect to the 2-Wasserstein metric. The equation has been studied as a model for the evolution of vortex densities in superconductors [28, 98] and as a model for adhesion dynamics [80, 85].

It is a straightforward calculation that any smooth solution to (1.1) conserves mass, so we can unambiguously write $M=\int _{{\mathbb {R}}^2}\theta (x)dx = \int _{{\mathbb {R}}^2}\theta ^0(x)dx$. Suppose that $\theta$ is a solution to (1.1) with finite second moment $\int _{{\mathbb {R}}^2}|x|^2\theta ^t(x)dx$. Evidently this quantity is strictly positive if $\theta ^t$ is not identically zero. Using integration by parts, one computes

1.2 $$\begin{aligned} \frac{d}{dt}\int _{{\mathbb {R}}^2}|x|^2\theta ^t(x)dx = -\frac{M^2}{2\pi } + 4\nu M = \frac{M}{2\pi }\left( 8\pi \nu -M\right) . \end{aligned}$$

Thus, if $M>8\pi \nu$, then the second moment is strictly decreasing at a linear rate. Since the second moment is nonnegative, this implies that the maximal time of existence for $\theta$ is finite. In particular, we see that if $\nu =0$, so there is no diffusion, then any nonzero, sufficiently localized classical solution to (1.1) must have finite lifespan [65]. In fact, for initial datum in $L^1$, one has a unique, global mild solution to (1.1) if and only if $M\le 8\pi \nu$ [97]. For the asymptotic behavior of solutions, we refer to [6, 20] ($M<8\pi \nu$), [4, 54] ($M=8\pi \nu$), and [94–96] ($M>8\pi \nu$), and references therein. In the case $\nu =0$, one has a sharp bound for the time of existence for compactly supported $L^\infty$ weak solutions to (1.1), which are necessarily unique, as well as exact solutions that provide an explicit example of finite-time collapse to a nontrivial measure [12].

For the deterministic dynamics of Eq. (1.1), we see that global existence is a nonstarter for classical solutions if the diffusion is too weak relative to the size of the initial data. But in the past two decades, there has been intense research activity on understanding how adding some noise structure (in varying forms) to deterministic equations can impact the behavior of solutions. A small, non-exhaustive sample of this research is given by [7, 14, 22, 36, 37, 41, 44–47, 50] and references therein. Concerning equations of the form (1.1), Flandoli et al. [43] have shown that blow-up is *delayed* in a 3D version of (1.1) with positive $\nu$ on ${\mathbb {T}}^d$ by adding a suitable multiplicative noise of transport type. Misiats et al. [77] have shown that some choices of random perturbations of Eq. (1.1) with $\nu >0$ lead to global solutions for small-mass initial data, while other choices lead to finite-time blow-up with positive probability for all initial data .

To the best of our knowledge, prior works have not shown that noise prevents finite-time blow-up, in particular for the case $\nu =0$ when all smooth, sufficiently localized solutions necessarily blow up in finite time.^1^ Recently, the second author together with Buckmaster et al. [14] showed that adding *random diffusion* leads to global solutions with positive probability for the invsicid surface quasi-geostrophic (SQG) equation with Gevrey-type initial data. Unlike the Eq. (1.1), inviscid SQG is a Hamiltonian flow, and the long-time dynamics of classical solutions is still unresolved. In light of this result, it is natural to ask if random diffusion may somehow improve the existence theory for the Eq. (1.1), for which solutions a priori behave very differently. Thus, we pose the following question, which the present article seeks to answer.

#### Question 1.1

Can one restore global existence of sufficiently regular solutions to (1.1) by adding a suitable *random diffusion*?

### Problem formulation

In order to investigate the regularizing effect of random diffusion and answer Question 1.1, let us start from more general deterministic equations of the form

1.3 $$\begin{aligned} {\left\{ \begin{array}{ll} {\partial }_t\theta + {{\,\mathrm{\textrm{div}}\,}}\left( \theta {\mathbb {M}}\nabla {\textsf{g}}*\theta \right) = 0 \\ \theta |_{t=0} = \theta ^0 \end{array}\right. } \qquad (t,x) \in {\mathbb {R}}\times {\mathbb {R}}^d. \end{aligned}$$

One could also include a diffusion term $-|\nabla |^\lambda \theta$, for $\lambda >0$, in the right-hand side (see Remark 1.8 below), but we will not do so here. Above, ${\mathbb {M}}$ is a $d\times d$ constant matrix. There are several meaningful choices for ${\mathbb {M}}$. For instance, if we choose ${\mathbb {M}}$ to be $-\mathbb {I}$, then we get gradient flows. While if we choose ${\mathbb {M}}$ to be antisymmetric,^2^ then we obtain conservative/Hamiltonian flows. We assume that ${\textsf{g}}\in {\mathcal {S}}'({\mathbb {R}}^d)$ is a tempered distribution, such that the Fourier transform of $\nabla {\textsf{g}}$ is locally integrable and satisfies the bound $|\xi {\hat{{\textsf{g}}}}(\xi )| \lesssim |\xi |^{1-\gamma }$ for some $0<\gamma <d+1$. The model case is when ${\textsf{g}}$ is a log or Riesz potential according to the rule

1.4 $$\begin{aligned} \pm {\left\{ \begin{array}{ll} -\log |x|, &{} {\gamma = d} \\ |x|^{\gamma -d}, &{} {\gamma \in (0,d+1)\setminus \{d\}}. \end{array}\right. } \end{aligned}$$

The choice of sign determines whether the potential is repulsive ($+$) or attractive (−). When $\gamma =2$, (1.4) is a constant multiple of the Coulomb/Newtonian potential. We refer to the ranges $\gamma >2$ and $\gamma <2$ as sub-Coulombic and super-Coulombic, respectively.

The general equation (1.3) encompasses a wide class of physical models. Focusing first on the conservative case, in which ${\mathbb {M}}$ is antisymmetric, the most notable examples are in dimension 2. If ${\mathbb {M}}$ is rotation by $\frac{\pi }{2}$ and ${\textsf{g}}(x)=-\frac{1}{2\pi }\log |x|$ is the Coulomb potential, then (1.3) becomes the incompressible Euler vorticity equation (for instance, see [76, Section 1.2] or [73, Chapter 2]). If ${\textsf{g}}(x)=C|x|^{-1}$, then Eq. (1.3) becomes the inviscid SQG equation, which models the motion of a rotating stratified fluid with small Rosby and Froude numbers in which potential vorticity is conserved [26, 62, 83, 87]. More generally, choosing ${\textsf{g}}(x) = C_\gamma |x|^{\gamma -2}$, for $0<\gamma <2$, leads to the generalized SQG (gSQG) family of equations [18, 84], for which the Euler vorticity equation is the $\gamma \rightarrow 2^-$ limit. While global-well posedness is known for classical [58, 99] and weak [100] solutions to the Euler case, the global existence of smooth solutions to the gSQG equation is a major open problem—it is only known if one adds suitably strong diffusion to (1.3) (e.g. see [30, 33, 35, 67]). We refer the reader to [16, 17, 21, 23, 26, 49, 57, 60, 87] and references therein for more information on the well-posedness and long-time dynamics of the gSQG equation.

In the gradient-flow case, in which ${\mathbb {M}}=-\mathbb {I}$, Eq. (1.3) has been studied for several applications in addition to the aforementioned ones of adhesion dynamics, chemotaxis, and vortices in superconductors. To name a few: materials science [61], cooperative control [56], granular flow [3, 5, 27, 93], phase segregation in lattice matter models [51–53], and swarming models [74, 75, 91, 92]. Several works have focused on the well-posedness and long-time dynamics. We recount some of the results for the model interaction (1.4), which is sometimes called a fractional porous medium equation. In particular, in the repulsive case $\gamma =2$, global existence, uniqueness, and asymptotic behavior of nonnegative classical and $L^\infty$ weak solutions are known [2, 12, 71, 89]. The case $2<\gamma < d+1$ is easier and follows by the same arguments [19, Section 4] (see also [13] for an $L^p$ well-posedness result). For $0<\gamma <2$, local well-posedness of nonnegative classical solutions is known [25] and global existence, regularity, and asymptotic behavior of certain nonnegative weak solutions are known [9, 24, 29, 31, 32, 34, 70]. To our knowledge, these weak solutions are only known to be unique if $d=1$ [11]. It is also an open problem whether classical solutions are global if $0<\gamma <2$. If one allows for mixed-sign solutions, then the repulsive and attractive equations are equivalent by multiplication by $-1$. [12] has established well-posedness, in particular maximal time of existence, for compactly supported classical and $L^\infty$ weak solutions in the $\gamma =2$ case. In particular, nonnegative classical and $L^\infty$ weak solutions in the $\gamma =2$ case are known to blow up in finite time, as remarked at the beginning of the introduction. [78] has shown the existence of global renormalized solutions in the sense of DiPerna–Lions [38, 39]. We also mention the works [1, 72, 78] for an equation arising in vortex superconductivity, which reduces to the repulsive $\gamma =2$ case of Eq. (1.3) when one considers nonnegative solutions.

As a unifying perspective, the Eq. (1.3) may be viewed as an effective description of first-order mean-field dynamics of the form

1.5 $$\begin{aligned} {\left\{ \begin{array}{ll} {\dot{x}}_i^t = \displaystyle \frac{1}{N}\sum _{1\le j\le N : j\ne i} {\mathbb {M}}\nabla {\textsf{g}}(x_i^t-x_j^t) \\ x_i^t|_{t=0} = x_i^0 \end{array}\right. } \qquad i\in \{1,\ldots ,N\} \end{aligned}$$

in the limit as the number of particles $N\rightarrow \infty$. The mathematical validity of this description has been actively studied over the years [10, 15, 19, 40, 42, 55, 59, 64, 66, 81, 88, 90].

Inspired by the aforementioned work of Buckmaster et al. [14], which in turn was inspired by earlier work of Glatt-Holtz and Vicol [50], we propose adding a random diffusion term to (1.3) by considering the stochastic partial differential equation

1.6 $$\begin{aligned} {\left\{ \begin{array}{ll} {\partial }_t\theta +{{\,\mathrm{\textrm{div}}\,}}(\theta {\mathbb {M}}\nabla {\textsf{g}}*\theta ) = \nu (1+|\nabla |^s)\theta {\dot{W}}^t \\ \theta |_{t=0} = \theta ^0 \end{array}\right. } \qquad (t,x)\in {\mathbb {R}}\times {\mathbb {R}}^d. \end{aligned}$$

Here, $s\ge 0$ (we will determine further restrictions later), *W* is a standard real Brownian motion, and the stochastic differential should be interpreted in the Itô sense. The $\dot{}$ superscript formally denotes differentiation with respect to time. We note that our choice of random diffusion differs from that of [14], which used the fractional Laplacian $|\nabla |^s = (-\Delta )^{\frac{s}{2}}$. In that article, the authors work in the periodic setting of ${\mathbb {T}}^2$, and after modding out by the mass of the solution, which is conserved, homogeneous and inhomogeneous Sobolev spaces are equivalent. This equivalence fails on ${\mathbb {R}}^d$, and therefore the fractional Laplacian creates problems at low frequency, as will become clear to the reader in Sects. 3 and 4 (see Remark 1.7 for further comments). Accordingly, we opt to add an inhomogeneity to rectify this issue. We emphasize that our choice of random perturbation differs from the aforementioned prior works [43, 77] on stochastic PKS equations, which did *not* consider random diffusion as in (1.6).

A priori, it is not clear how to interpret the SPDE (1.6). Moreover, it is not clear that the stochastic term in the right-hand side is regularizing since $W^t$ does not have definite sign. Formally, suppose that we have a solution $\theta$ to (1.6), and let us set $\mu ^t:=e^{-\nu W^t(1+|\nabla |^s)}\theta ^t$, where for each realization of *W*, $\Gamma ^t:=e^{-\nu W^t(1+|\nabla |^s)}$ is the Fourier multiplier with symbol $e^{-\nu W^t(1+|\xi |^s)}$. As in [14], to compute the equation satisfied by $\mu$, we formally use the Fourier transform together with Itô’s lemma to obtain

1.7 $$\begin{aligned} {\partial }_t\mu&= \Gamma {\partial }_t\theta + {\partial }_t\Gamma \theta + {\partial }_t[{\Gamma }{\theta }] \nonumber \\&= \Gamma \left( -{{\,\mathrm{\textrm{div}}\,}}(\theta {\mathbb {M}}\nabla {\textsf{g}}*\theta ) + \nu (1+|\nabla |^s)\theta {\dot{W}}\right) \nonumber \\&\quad + \left( -\nu (1+|\nabla |^s)\Gamma {\dot{W}}+ \frac{\nu ^2}{2}(1+|\nabla |^{s})^2\Gamma \right) \theta -\nu ^2(1+|\nabla |^{s})^2\Gamma \theta \nonumber \\&= - {{\,\mathrm{\textrm{div}}\,}}\Gamma \left( \Gamma ^{-1}\mu {\mathbb {M}}\nabla {\textsf{g}}*\Gamma ^{-1}\mu \right) - \frac{\nu ^2}{2}(1+|\nabla |^{s})^2\mu . \end{aligned}$$

Above, $[\Gamma ,\theta ]$ denotes the quadratic covariation of the processes $\Gamma$ and $\theta$. Also, we have implicitly used that $\Gamma ^t$ and $|\nabla |^s$ commute, both being Fourier multipliers. Observe that (1.7) is a random PDE which may be interpreted pathwise (i.e. for fixed realization of *W*, which almost surely is a locally continuous path on $[0,\infty )$). Additionally, thanks to the nontrivial quadratic variation of Brownian motion, we have gained a diffusion term in this equation. Rather than deal with the original equation (1.6), we shall base our mathematical interpretation on (1.7).

#### Remark 1.1

One may wonder why we choose the Itô formulation in (1.6) as opposed to the Stratonovich formulation

1.8 $$\begin{aligned} {\partial }_t\theta +{{\,\mathrm{\textrm{div}}\,}}(\theta {\mathbb {M}}\nabla {\textsf{g}}*\theta ) = \nu (1+|\nabla |^s)\theta \circ {\dot{W}}^t \end{aligned}$$

which is formally equivalent to the Itô equation

1.9 $$\begin{aligned} {\partial }_t\theta + {{\,\mathrm{\textrm{div}}\,}}(\theta {\mathbb {M}}\nabla {\textsf{g}}*\theta ) = \nu (1+|\nabla |^s)\theta {\dot{W}}^t + \frac{\nu ^2}{2}(1+|\nabla |^{s})^2\theta . \end{aligned}$$

Suppose we define $\mu ^t :=e^{-\nu W^t(1+|\nabla |^s)}\theta ^t$ as before. Then again using Itô’s lemma, we find

1.10 $$\begin{aligned} {\partial }_t\mu&= \Gamma {\partial }_t\theta + {\partial }_t\Gamma \theta + {\partial }_t[{\Gamma }{\theta }] \nonumber \\&=\Gamma \left( -{{\,\mathrm{\textrm{div}}\,}}(\theta {\mathbb {M}}\nabla {\textsf{g}}*\theta ) + \nu (1+|\nabla |^s)\theta {\dot{W}}^t + \frac{\nu ^2}{2}(1+|\nabla |^{s})^2\theta \right) \nonumber \\&\quad + \left( -\nu (1+|\nabla |^s)\Gamma {\dot{W}} + \frac{\nu ^2}{2}(1+|\nabla |^{s})^2\Gamma \right) \theta -\nu ^2(1+|\nabla |^{s})^2\Gamma \theta \nonumber \\&= - \Gamma {{\,\mathrm{\textrm{div}}\,}}(\Gamma ^{-1}\mu {\mathbb {M}}\nabla {\textsf{g}}*\Gamma ^{-1}\mu ). \end{aligned}$$

No longer do we gain a fractional diffusion term, which, as we shall see below, is fatal to our arguments. The preceding conclusion is to be expected. Indeed, if *W* is a $C^1$ path, then by ordinary calculus, ${\partial }_t\mu = -\nu (1+|\nabla |^s)\Gamma \theta {\dot{W}} + \Gamma {\partial }_t\theta$; and the Stratonovich formulation is precisely chosen to preserve the ordinary rules of calculus.

### Statement of main results

We now present our main theorem. We assume that we have a standard real Brownian motion $\{W^t\}_{t\ge 0}$ defined on a filtered probability space $(\Omega , {\mathcal {F}}, \{{\mathcal {F}}^t\}_{t\ge 0},\mathcal {P})$ satisfying all the usual assumptions. Given $\alpha ,\beta ,\nu >0$, consider the event

1.11 $$\begin{aligned} \Omega _{\alpha ,\beta ,\nu } :=\{\omega \in \Omega : \alpha +\beta t - \nu W^t(\omega ) \ge 0 \quad \forall t\in [0,\infty )\} \subset \Omega . \end{aligned}$$

It is well-known (see [86, Proposition 6.8.1]) that $\mathcal {P}(\Omega _{\alpha ,\beta ,\nu }) = 1 - e^{-\frac{2\alpha \beta }{\nu ^2}}$. We recall the Fourier–Lebesgue norms $\Vert \cdot \Vert _{{\hat{W}}^{\kappa ,r}}$ (see Sect. 2.2).

#### Theorem 1.2

Let $d\ge 1$, $0<\gamma <d+1$, $\frac{1}{2}<s\le 1$. If $\gamma >1$, also suppose that we are given $1<q<\frac{d}{\gamma -1}$. Given $\alpha ,\beta ,\nu >0$, suppose that $\beta <\frac{\nu ^2}{2}$.

Suppose first that $\gamma \le 1$. If *s* is sufficiently large depending on $\gamma$, then there exists an $r_0\ge 1$ depending on $d,\gamma ,s$, such that the following holds. For any $1\le r \le r_0$ and any $\sigma >0$ sufficiently large depending on $d,\gamma ,r,s$, there is a constant $C>0$ depending only on $d,\gamma ,r,s,\sigma$, such that for initial datum $\mu ^0$ satisfying

1.12 $$\begin{aligned} \Vert e^{(\alpha +\epsilon )(1+|\nabla |^{s})}\mu ^0\Vert _{{\hat{W}}^{\sigma s,r}} < \frac{\nu ^2-2\beta }{C|{\mathbb {M}}|} \end{aligned}$$

and any path in $\Omega _{\alpha ,\beta ,\nu }$, there exists a unique global solution $\mu \in C([0,\infty ); {\hat{W}}^{\sigma s,r})$ to Eq. (1.7) with initial datum $\mu ^0$. Moreover, for $\phi ^t:=\alpha +\beta t$ and assuming $\mu ^0\ne 0$,^3^ the function

1.13 $$\begin{aligned} t\mapsto \Vert e^{(\phi ^t+\epsilon )(1+|\nabla |^s)}\mu ^t\Vert _{{\hat{W}}^{\sigma s,r}} \end{aligned}$$

is strictly decreasing on $[0,\infty )$.

Now suppose that $\gamma >1$. For $\sigma _r,\sigma _q>0$ sufficiently large depending on $d,\gamma ,s,r,q$, there is a constant $C>0$ depending only on $d,\gamma ,r,q,s,\sigma _r,\sigma _q$, such that for initial datum $\mu ^0$ satisfying

1.14 $$\begin{aligned} \Vert e^{(\alpha +\epsilon )(1+|\nabla |^{s})}\mu ^0\Vert _{{\hat{W}}^{\sigma _r s,r}} + \Vert e^{(\alpha +\epsilon )(1+|\nabla |^{s})}\mu ^0\Vert _{{\hat{W}}^{\sigma _q s,\frac{2q}{q-1}}} < \frac{\nu ^2-2\beta }{C|{\mathbb {M}}|} \end{aligned}$$

and any path in $\Omega _{\alpha ,\beta ,\nu }$, there exists a unique global solution $\mu \in C([0,\infty ); {\hat{W}}^{\sigma _r s,r} \cap {\hat{W}}^{\sigma _q s, q})$ to Eq. (1.7) with initial datum $\mu ^0$. Moreover, assuming $\mu ^0\ne 0$, the function

1.15 $$\begin{aligned} t\mapsto \Vert e^{(\phi ^t+\epsilon )(1+|\nabla |^s)}\mu ^t\Vert _{{\hat{W}}^{\sigma _r s,r}} + \Vert e^{(\phi ^t+\epsilon )(1+|\nabla |^s)}\mu ^t\Vert _{{\hat{W}}^{\sigma _q s,\frac{2q}{q-1}}} \end{aligned}$$

is strictly decreasing on $[0,\infty )$.

To the best our knowledge, our result is the first demonstration that a random diffusion term can lead to global solutions for equations which, without any diffusion, necessarily blow up in finite time. This provides an affirmative answer to Question 1.1. Furthermore, Theorem 1.2 substantially generalizes the prior work of Buckmaster et al. [14, Theorem 1.1], which was limited to the SQG case ${\mathbb {M}}$ equals rotation by $\frac{\pi }{2}$ and ${\hat{{\textsf{g}}}}(\xi ) = |\xi |^{-1}$, corresponding to a conservative/Hamiltonian flow. In particular, our result covers the full range of interactions in the model case (1.4) and also allows for interactions (e.g. $d<\gamma <d+1$) which may not be singular in physical space near the origin but have very slow decay or even growth at $\infty$.

We do not say anything here about the asymptotic behavior of the solutions we construct, only that they are global. It would be interesting to give an asymptotic description of the solution as $t\rightarrow \infty$, valid at least with positive probability. Indeed, the reader will recall from the beginning of the introduction that such a description is known for the deterministic PKS equation. We hope to address this question in future work.

Before transitioning to discuss the proof of Theorem 1.2, let us record a few remarks on the statement of and assumptions behind the theorem.

#### Remark 1.3

The solutions in Theorem 1.2 are *pathwise*. More precisely, there is a good set $\Omega _{\alpha ,\beta ,\nu }$, defined above, of realizations of the Brownian motion, such that for any $\omega \in \Omega _{\alpha ,\beta ,\nu }$ and with $W(\omega ): [0,\infty )\rightarrow {\mathbb {R}}$, we have a unique global solution $\mu (\omega ): [0,\infty ) \rightarrow {\mathbb {R}}$ to Eq. (1.7). We can then define a notion of solution to the original equation (1.6) by setting $\theta ^t(\omega ) :=e^{\nu W^t(\omega )(1+|\nabla |^s)}\mu ^t(\omega )$. Since for any $\omega \in \Omega _{\alpha ,\beta ,\nu }$, we have $\phi ^t - \nu W^t\ge 0$, it follows from the definition of the Fourier–Lebesgue norm that

1.16 $$\begin{aligned} \Vert \theta ^t(\omega )\Vert _{{\hat{W}}^{\sigma s,p}} \le \Vert e^{\phi ^t(1+|\nabla |^s)}\mu ^t(\omega )\Vert _{{\hat{W}}^{\sigma s,p}}. \end{aligned}$$

for any $$1\le p\le \infty $$.

One may interpret Theorem 1.2 as follows (cf. [14, Remark 5.3]). Fixing $\alpha ,\epsilon ,\nu$ and given an initial condition $\mu ^0$ such that

1.17 $$\begin{aligned} E:=\Vert e^{(\alpha +\epsilon )(1+|\nabla |^s)}\mu ^0\Vert _{{\hat{W}}^{\sigma _r s,r}} + \Vert e^{(\alpha +\epsilon )(1+|\nabla |^s)}\mu ^0\Vert _{{\hat{W}}^{\sigma _q s,\frac{2q}{q-1}}}\textbf{1}_{\gamma >1} < \infty , \end{aligned}$$

we can choose $\beta < \frac{\nu ^2-CE|{\mathbb {M}}|}{2}$, where *C* is the constant from condition (1.12) or (1.14). Then with probability at least

1.18 $$\begin{aligned} P = 1- \exp \left( -\frac{\alpha (\nu ^2-CE|{\mathbb {M}}|)}{\nu ^2}\right) , \end{aligned}$$

there is a pathwise unique global solution $\mu \in C([0,\infty ); {\hat{W}}^{\sigma _r s, r} \cap {\hat{W}}^{\sigma _q s, \frac{2q}{q-1}})$ to Eq. (1.7) with initial datum $\mu ^0$, such that the function (1.15) is strictly decreasing on $[0,\infty )$.

#### Remark 1.4

So as to make the result as accessible as possible, we have opted not to include in the statement of Theorem 1.2 the explicit relations the parameters, such as $d,\gamma ,s,r_0,\sigma$, have to satisfy in order for the theorem to apply. These relations are explicitly worked out in Sects. 3 and 4 during the proofs of Propositions 3.1 and 4.1. Here and throughout this article, the reader should keep in mind that the most favorable choices for *s*, *r* are $s=1$ and $r=1$.

#### Remark 1.5

The conditions (1.12), (1.14) allows for initial data of arbitrarily large mass. Indeed, focusing on the $\gamma \le 1$ case, suppose that ${\hat{\mu }}^0\in C_c^\infty$ and ${\hat{\mu }}^0(0) = M$, for given *M*. Let $L= \sup _{\xi \in {{\,\textrm{supp}\,}}({\hat{\mu }}^0)} |\xi |$. Then

1.19 $$\begin{aligned} \Vert e^{(\alpha +\epsilon )(1+|\nabla |^s)}\mu ^0\Vert _{{\hat{W}}^{\sigma s,r }} \le C_d\left( 1+L\right) ^{\sigma s} L^{\frac{d}{r}}e^{(\alpha +\epsilon )(1+L^s)}\Vert {\hat{\mu }}^0\Vert _{L^\infty }. \end{aligned}$$

Taking $\beta = \varepsilon \frac{\nu ^2}{2}$, for given $\varepsilon \in (0,1)$, and requiring

1.20 $$\begin{aligned} \frac{C_dC|{\mathbb {M}}|\left( 1+L\right) ^{\sigma s} L^{\frac{d}{r}}e^{(\alpha +\epsilon )(1+L^s)}\Vert {\hat{\mu }}^0\Vert _{L^\infty }}{(1-\varepsilon )} < \nu ^2, \end{aligned}$$

we see that (1.12) holds. For fixed $\nu$, we can make the left-hand side of the preceding inequality arbitrarily small by letting $L\rightarrow 0^+$. While for given *L*, we can take $\nu$ arbitrarily large so that (1.20) holds. The latter case is reminiscent of the mass-diffusion threshold we saw earlier for the PKS equation.

#### Remark 1.6

Although only the ratio $\frac{2\beta }{\nu ^2}$ appears in the value of $\mathcal {P}(\Omega _{\alpha ,\beta ,\nu })$, which might suggest to the reader that one can send $\beta ,\nu \rightarrow 0^+$ while fixing $\frac{2\beta }{\nu ^2}$, we emphasize that the assumption $0<\beta <\frac{\nu ^2}{2}$ is crucial. Indeed, our argument for showing local well-posedness fails if $\beta \ge \frac{\nu ^2}{2}$, and the requirement $\beta <\frac{\nu ^2}{2}$ appears when showing the functions (1.13), (1.15) are strictly decreasing. Furthermore, if $\nu ,\beta \rightarrow 0^+$, then the right-hand sides of the initial datum conditions (1.12), (1.14) are tending to zero, which implies that only $\mu ^0\equiv 0$ would satisfy these conditions in the limit.

Additionally, one might think that by increasing $\nu$, the diffusion becomes stronger and therefore one should get a “better” result. But $\mathcal {P}(\Omega _{\alpha ,\beta ,\nu })$ evidently decreases to zero as $\nu \rightarrow \infty$, assuming $\beta$ is fixed. The reason has to deal with the resulting growing variance of $\nu W^t$ appearing in the definition of $\Gamma ^t$, which requires a large value of $\beta$ to be absorbed by the exponential weight in our function spaces.

#### Remark 1.7

Theorem 1.2 is also valid if ${\mathbb {R}}^d$ is replaced by ${\mathbb {T}}^d$. In fact, since Fourier space is discrete on the torus, we do not have the same issues at low frequency as in the setting of ${\mathbb {R}}^d$, and therefore one can replace Eq. (1.6) with

1.21 $$\begin{aligned} {\partial }_t\theta + {{\,\mathrm{\textrm{div}}\,}}(\theta {\mathbb {M}}\nabla {\textsf{g}}*\theta ) = \nu |\nabla |^s\theta {\dot{W}}^t. \end{aligned}$$

An elementary computation reveals that solutions conserve mass and therefore one may quotient out the mass by assuming it is zero. As a result, the zero Fourier mode vanishes and one has an equivalence of homogeneous and inhomogeneous Sobolev norms. Working with (1.21) simplifies the proof greatly, as the two-tiered norm for $\gamma >1$ becomes unnecessary.

#### Remark 1.8

Theorem 1.2 is still valid if one adds a deterministic diffusion term $-\chi |\nabla |^{\lambda }\theta$ to the right-hand side of (1.6), for $\chi ,\lambda >0$, which leads to (1.7) being replaced by

1.22 $$\begin{aligned} {\partial }_t\mu + {{\,\mathrm{\textrm{div}}\,}}\Gamma \left( \Gamma ^{-1}\mu {\mathbb {M}}\nabla {\textsf{g}}*\Gamma ^{-1}\mu \right) = -\left( \frac{\nu ^2}{2}(1+|\nabla |^{s})^2 + \chi |\nabla |^{\lambda }\right) \mu . \end{aligned}$$

Since a deterministic diffusion term only makes the circumstances for global existence more favorable, we have opted not to include this term.

### Comments on proof

We briefly comment on the proof of Theorem 1.2. In light of the success of [14] in showing that adding random diffusion to the inviscid SQG equation leads, with high probability, to global solutions, and that the SQG equation is a special case of (1.3), we are guided by the approach of the cited work. There are two main steps:

1. Local well-posedness via contraction mapping argument,
2. Monotonicity of the Gevrey norm via energy estimate.

As discussed below, repeating the proof of [14] in our more general context would fail due to issues at low frequency related to working on ${\mathbb {R}}^d$, as opposed to ${\mathbb {T}}^d$, and issues at high frequency stemming from the singularity of our interactions. Several new ideas are consequently needed.

Step (1), carried out in Sect. 3, proceeds by rewriting the Eq. (1.7) in mild form (see (3.3)) which is amenable to a contraction mapping argument for short times. The main difficulty is estimating the nonlinear term in the scale of Gevrey-type spaces defined in Sect. 3.1—the exponential weights of which are used to absorb the $\Gamma$ operators—in which we want to construct solutions. In particular, the velocity field ${\mathbb {M}}\nabla {\textsf{g}}*\mu$ can be singular compared to the regularity of the scalar $\mu$, as opposed to of the same order in the SQG case of [14], which requires carefully balancing the derivatives in the nonlinearity against the diffusion.

It turns out that using $L^2$-based function spaces, as in [14], leads to a restriction on $\gamma$ that scales linearly in the dimension *d*, which would then limit us to strictly sub-Coulombic interactions ${\textsf{g}}$ in dimensions $d\ge 4$. One of our new insights is to instead consider Gevrey–Fourier–Lebesgue hybrid spaces (see (3.4)), which of course include the function spaces of [14] as a special case. In particular, our new spaces behave well with respect to Sobolev embedding when the integrability exponent $r\rightarrow 1^+$, becoming an algebra at $r=1$.

Another challenge in the local well-posedness step is the singularity near the origin of the Fourier transform ${\hat{{\textsf{g}}}}$ when $\gamma$ is large. In particular, for $\gamma >1$, $|\xi {\hat{{\textsf{g}}}}(\xi )|$ may blow up as $|\xi |\rightarrow 0$. Dealing with this issue requires using a two-tiered function space, compared to the case $\gamma \le 1$. More precisely, at high frequency, we need our functions to be in an exponentially-weighted Fourier–Lebesgue space with high regularity index and low integrability exponent; while at low frequency, we need our functions to be in a similarly weighted space with low regularity index and high integrability exponent. This leads us to the multi-parameter scale of spaces $\textsf{X}_{\phi ,\gamma }^{\sigma ,\tilde{\sigma },r,\tilde{r},}$ introduced in (3.7) (more generally, see Sect. 3).

After some paraproduct analysis and a fair amount of algebra to determine what conditions all the various parameters have to satisfy, we prove Proposition 3.1, which asserts local well-posedness in the class of solutions satisfying

1.23 $$\begin{aligned} \sup _{0\le t\le T} \left( \Vert e^{(\phi ^t+\epsilon )(1+|\nabla |^s)}\mu ^t\Vert _{{\hat{W}}^{\sigma _{lwp},r}} + \Vert e^{(\phi ^t+\epsilon )(1+|\nabla |^s)}\mu ^t\Vert _{{\hat{W}}^{0,\frac{2q}{q-1}}}\textbf{1}_{\gamma >1}\right) < \infty , \end{aligned}$$

for some $\sigma _{lwp} < \sigma$. Here, $\textbf{1}_{(\cdot )}$ denotes the indicator function for the condition $(\cdot )$. Although the Sobolev index $\sigma _{lwp}$ is strictly less than that of the initial datum, we will later improve it to $\sigma$ through a bootstrap argument.

Step (2), carried out in Sect. 4, consists of upgrading the local solution from step (1) to a global solution and also upgrading the Sobolev index from $\sigma _{lwp}$ to $\sigma$. The original idea of [14, Proposition 4.1], modified to our setting and presented for the $r=2$ case, is to prove an inequality for the time derivative of the “energy” $\Vert e^{\phi ^t(1+|\nabla |^s)}\mu ^t\Vert _{H^{\sigma s}}^2$, which shows that this quantity is strictly decreasing on an interval [0, *T*], provided it is not too large at initial time and that $\Vert e^{\phi ^t(1+|\nabla |^s)}\mu ^t\Vert _{H^{(\sigma +1)s}}$ remains finite on the same interval. With this type of conditional monotonicity result, the authors of that work could exploit the fact that the initial datum belongs to a space with higher Gevrey index $\alpha +\epsilon$ in order to iteratively extend the lifespan of the solution, losing a decreasing fraction of $\epsilon$ along each step of the iteration. Note that in their work the Sobolev index from the local well-posedness does not change.

Since we deal with $\gamma$ that are more singular than in [14] and step (1) only gives local solutions in a rougher space than that claimed in the statement of Theorem 1.2, we need a more sophisticated argument. Moreover, we need to work in our scale of Fourier–Lebesgue spaces, with the auxiliary space if $\gamma >1$. We prove a similar conditional monotonicity result for the energy

1.24 $$\begin{aligned} \Vert e^{(\phi ^t+\epsilon ')(1+|\nabla |^s)}\mu ^t\Vert _{{\hat{W}}^{\sigma _{r} s,r}}^r + \Vert e^{(\phi ^t+\epsilon ')(1+|\nabla |^s)}\mu ^t\Vert _{{\hat{W}}^{\sigma _{q} s,\frac{2q}{q-1}}}^{\frac{2q}{q-1}}\textbf{1}_{\gamma >1}, \end{aligned}$$

for any $0\le \epsilon '\le \epsilon$, assuming $\sigma _{r},\sigma _{q}$ are sufficiently large depending on $d,\gamma ,s,r,q$. Similar to step (1), the bulk of the labor consists of paraproduct analysis for the nonlinearity and determining the set of conditions that the parameters $d,\gamma ,s,r,q,\sigma _{r},\sigma _{q}$ have to satisfy in order for the paraproduct analysis to be valid. In order to access the monotonicity result, since $\sigma _r > \sigma _{lwp}$ and $\sigma _q>0$, we exploit the higher Gevrey index of the initial datum together with an embedding lemma (see Lemma 3.5) to conclude that if (1.23) holds, then for any $0\le \epsilon '<\epsilon$ and $\sigma _r,\sigma _q\in {\mathbb {R}}$,

1.25 $$\begin{aligned} \sup _{0\le t\le T}\left( \Vert e^{(\phi ^t + \epsilon ')(1+|\nabla |^s)}\mu ^t\Vert _{{\hat{W}}^{\sigma _r s,r}} + \Vert e^{(\phi ^t+\epsilon ')(1+|\nabla |^s)}\mu ^t\Vert _{{\hat{W}}^{\sigma _q s,\frac{2q}{q-1}}}\right) < \infty \nonumber \\ \end{aligned}$$

also holds. We then obtain global existence by a lemma (see Lemma 4.5) which quantifies the improvement in the lifespan of the solution as we decrease $\epsilon '$. Finally, we conclude global existence and monotonicity also hold with $\epsilon '=\epsilon$ by essentially monotone convergence theorem.

### Organization of article

We close the introduction by outlining the remaining body of the article. In Sect. 2, we introduce the basic notation of the article and review some frequently used facts from Fourier analysis. In Sect. 3, we begin (Sect. 3.1) with our class of Gevrey–Fourier–Lebesgue spaces and their properties and then (Sect. 3.2) show the local well-posedness of the Cauchy problem for Eq. (1.7). In Sect. 4, we first (Sect. 4.1) show the monotonicity property of the Gevrey norm. We then (Sect. 4.2) use this property together with the local theory from Sect. 3 in order to prove our main result, Theorem 1.2.

## Preliminaries

### Notation

Given nonnegative quantities *A* and *B*, we write $A\lesssim B$ if there exists a constant $C>0$, independent of *A* and *B*, such that $A\le CB$. If $A \lesssim B$ and $B\lesssim A$, we write $A\sim B$. To emphasize the dependence of the constant *C* on some parameter *p*, we sometimes write $A\lesssim _p B$ or $A\sim _p B$. We denote the natural numbers excluding zero by ${\mathbb {N}}$ and including zero by ${\mathbb {N}}_0$. Similarly, we denote the positive real numbers by ${\mathbb {R}}_+$.

The Fourier and inverse transform of a function $f:{\mathbb {R}}^d\rightarrow {\mathbb {C}}^m$ are defined according to the convention

2.1 $$\begin{aligned} \begin{aligned} {\hat{f}}(\xi ) = {\mathcal {F}}(f)(\xi )&:=\int _{{\mathbb {R}}^d}f(x)e^{-ix\cdot \xi }dx,\\ {\check{f}}(x) = {\mathcal {F}}^{-1}(f)(x)&:=(2\pi )^{-d}\int _{{\mathbb {R}}^d}f(\xi )e^{i\xi \cdot x}d\xi . \end{aligned} \end{aligned}$$

In the case $m>1$, the notation should be understood component-wise. Given a function $m:{\mathbb {R}}^d\rightarrow {\mathbb {C}}^m$, we use the notation $m(\nabla )$ to denote the ${\mathbb {C}}^m$-valued Fourier multiplier with symbol $m(\xi )$. In the particular, the notation $|\nabla |= (-\Delta )^{\frac{1}{2}}$ denotes the Fourier multiplier with symbol $|\xi |$ and $\langle \nabla \rangle :=(1+|\nabla |^2)^{1/2}$ denotes the multiplier with Japanese bracket symbol $(1+|\xi |^2)^{1/2}$.

### Sobolev embedding

For the reader’s convenience, we state and prove an elementary Sobolev embedding tailored to the Fourier analysis of Sects. 3 and 4. To the state the lemma, we recall that the Bessel potential space $W^{s,p}$ is defined by

2.2 $$\begin{aligned} \Vert f\Vert _{W^{s,p}} :=\Vert \langle \nabla \rangle ^{s}f\Vert _{L^p}, \qquad s\in {\mathbb {R}}, \ p\in (1,\infty ) \end{aligned}$$

and the Fourier–Lebesgue space ${\hat{W}}^{s,p}$ is defined by

2.3 $$\begin{aligned} \Vert f\Vert _{{\hat{W}}^{s,p}} :=\Vert \langle \cdot \rangle ^s{\hat{f}}\Vert _{L^p}, \qquad s\in {\mathbb {R}}, \ p\in [1,\infty ]. \end{aligned}$$

For $p=2$, these two spaces coincide by Plancherel’s theorem, and, following standard notation, we shall write $H^s$. When $s=0$, we shall also adopt the notation ${\hat{W}}^{0,r} = {\hat{L}}^r$.

#### Lemma 2.1

If $$1\le p <r\le \infty $$, then

2.4 $$\begin{aligned} \Vert f\Vert _{{\hat{W}}^{s,p}} \lesssim _{d,p,r} \Vert f\Vert _{{\hat{W}}^{(s+\frac{d(r-p)}{rp})+,r}}, \end{aligned}$$

where the notation $(\cdot )+$ means $(\cdot )+\varepsilon$, for any $\varepsilon >0$, with the implicit constant then depending on $\varepsilon$ and possibly blowing up as $\varepsilon \rightarrow 0^+$. If $2\le p\le \infty$, then

2.5 $$\begin{aligned} \Vert f\Vert _{{\hat{W}}^{s,p}} \lesssim _{d,p} \Vert f\Vert _{W^{s,\frac{p}{p-1}}}. \end{aligned}$$

#### Proof

Fix $r> 1$ and let $1\le p<r$. By Hölder’s inequality,

2.6 $$\begin{aligned} \Vert f\Vert _{{\hat{W}}^{s,p}} = \Vert \langle \cdot \rangle ^{s}{\hat{f}}\Vert _{L^p} = \Vert \langle \cdot \rangle ^{-\delta }\langle \cdot \rangle ^{s+\delta }{\hat{f}}\Vert _{L^{p}} \lesssim _d \Vert \langle \cdot \rangle ^{-\delta }\Vert _{L^{\frac{rp}{r-p}}} \Vert f\Vert _{{\hat{W}}^{r,s+\delta }}. \end{aligned}$$

The first factor is finite provided that $\frac{r p\delta }{r-p}> d \Longleftrightarrow \delta > \frac{d(r-p)}{rp}$.

Now suppose $2 \le p\le \infty$. If $\langle \nabla \rangle ^s f\in L^{\frac{p}{p-1}}$, then by the Hausdorff–Young inequality $\mathcal {F}(\langle \nabla \rangle ^s f)(\xi ) = \langle \xi \rangle ^s {\hat{f}}(\xi )$ belongs to $L^p$ and

2.7 $$\begin{aligned} \Vert f\Vert _{{\hat{W}}^{s,p}} = \Vert \langle \cdot \rangle ^s {\hat{f}}\Vert _{L^{p}} \lesssim _{d,p} \Vert \langle \nabla \rangle ^s f\Vert _{L^{\frac{p}{p-1}}} = \Vert f\Vert _{W^{s,\frac{p}{p-1}}}. \end{aligned}$$

$$\square $$

## Local well-posedness

We investigate the local well-posedness of the Cauchy problem

3.1 $$\begin{aligned} {\left\{ \begin{array}{ll} {\partial }_t\mu + {{\,\mathrm{\textrm{div}}\,}}\Gamma \left( \Gamma ^{-1}\mu ({\mathbb {M}}\nabla {\textsf{g}}*\Gamma ^{-1}\mu )\right) + \frac{\nu ^2}{2}(1+|\nabla |^{s})^2\mu =0\\ \mu |_{t=0} = \mu ^0. \end{array}\right. } \end{aligned}$$

Set $A:=(1+|\nabla |^{s})^2$. It will be convenient to introduce the bilinear operator

3.2 $$\begin{aligned} B(f,g) :={{\,\mathrm{\textrm{div}}\,}}\Gamma \left( \Gamma ^{-1}f({\mathbb {M}}\nabla {\textsf{g}}*\Gamma ^{-1}g)\right) . \end{aligned}$$

The reader should note that *B* itself depends on time through $\Gamma$, and, when necessary, we shall make explicit this time dependence by writing $B^t(f,g)$. We rewrite (3.1) in mild form

3.3 $$\begin{aligned} \mu ^t = e^{-\frac{t\nu ^2}{2}A}\mu ^0 - \int _0^t e^{-\frac{(t-\tau )\nu ^2}{2}A} B^\tau (\mu ^\tau ,\mu ^\tau )d\tau . \end{aligned}$$

In order to perform a contraction mapping argument based on the mild formulation (3.3), we use a generalization of the scale of Gevrey function spaces from [14] (see also [48]). Given $a\ge 0$ and $\kappa \in {\mathbb {R}}$, we define

3.4 $$\begin{aligned} \Vert f\Vert _{{{\textsf{G}}}_a^{\kappa ,r}} :=\Vert e^{a A^{1/2}} f\Vert _{{\hat{W}}^{\kappa s,r}}. \end{aligned}$$

We refer to *a* as the *exponential weight* or *Gevrey index*, $\kappa$ as the *Sobolev index*, and *r* as the *integrability exponent*. If $r<\infty$, then the completion with respect to this norm of functions with compactly supported Fourier transforms in $L^r$ defines a Banach space, as the reader may check. For $0<T<\infty$ and a continuous function $\phi :[0,T]\rightarrow [0,\infty )$, we define

3.5 $$\begin{aligned} \Vert f\Vert _{C_T^0{{\textsf{G}}}_{\phi }^{\kappa ,r}} :=\sup _{0\le t\le T} \Vert f^t\Vert _{{\textsf{G}}_{\phi ^t}^{\kappa ,r}}. \end{aligned}$$

We write $C_{\infty }^0$ when $\sup _{0\le t\le T}$ is replaced by $\sup _{0\le t<\infty }$. Set

3.6 $$\begin{aligned} C_T^0{\textsf{G}}_{\phi }^{\kappa ,r} :=\{f\in C([0,T]; {\hat{W}}^{\kappa s,r}({\mathbb {R}}^d)) : \Vert f\Vert _{C_T^0{\textsf{G}}_{\phi }^{\kappa ,r}} < \infty \}. \end{aligned}$$

We also allow for $T=\infty$, replacing [0, *T*] in the preceding line with $[0,\infty )$. Evidently, this defines a Banach space.

To deal with possible issues at low frequencies when $\gamma$ is large, we also have need for the Banach spaces

3.7 $$\begin{aligned} \textsf{X}_{a,\gamma }^{\kappa _1,\kappa _2,r_1,r_2} :={\left\{ \begin{array}{ll} {\textsf{G}}_{a}^{\kappa _1,r_1}, &{} {\gamma \le 1} \\ {\textsf{G}}_{a}^{\kappa _1,r_1} \cap {\textsf{G}}_{a}^{\kappa _2,r_2}, &{} {\gamma > 1},\end{array}\right. } \end{aligned}$$

where

3.8 $$\begin{aligned} \Vert f\Vert _{{\textsf{G}}_{a}^{\kappa _1,r_1} \cap {\textsf{G}}_{a}^{\kappa _2,r_2}} :=\Vert f\Vert _{{\textsf{G}}_{a}^{\kappa _1,r_1} } + \Vert f\Vert _{{\textsf{G}}_{a}^{\kappa _2,r_2}}, \end{aligned}$$

and

3.9 $$\begin{aligned} C_T^0\textsf{X}_{\phi ,\gamma }^{\kappa _1,\kappa _2, r_1,r_2} :=\left\{ f\in C\left( [0,T]; {\hat{W}}^{\kappa _1 s, r_1}({\mathbb {R}}^d)\cap {\hat{W}}^{\kappa _2 s,r_2}({\mathbb {R}}^d)\right) : \Vert f\Vert _{C_T^0\textsf{X}_{\phi ,\gamma }^{\kappa _1,\kappa _2,r_1,r_2}}<\infty \right\} .\nonumber \\ \end{aligned}$$

The main result of this section is the following proposition.

### Proposition 3.1

Let $d\ge 1$, $0<\gamma <d+1$, $\frac{1}{2}<s\le 1$. If $\gamma >1$, then also suppose we are given $1\le q<\frac{d}{\gamma -1}$. Given $\alpha ,\beta >0$, suppose *W* is a realization from the set $\Omega _{\alpha ,\beta ,\nu }$ and that $\beta <\frac{\nu ^2}{2}$. Set $\phi ^t:=\alpha +\beta t$.

There exists $r_0 \ge 1$ depending on $d,\gamma ,s$, such that the following holds. For any $1\le r\le r_0$, there exists $\sigma _0 \in (0,\frac{2s-1}{s})$ depending on $d,\gamma ,r,s$, such that for any $\sigma \in (\sigma _0,\frac{2s-1}{s})$ with $1-\gamma \le \sigma s$, there exists a constant $C>0$ depending on $d,\gamma ,s,\sigma ,r,q,\beta ,\nu$, such that for $\Vert \mu ^0\Vert _{\textsf{X}_{\alpha ,\gamma }^{\sigma ,0,r,\frac{2q}{q-1}}} \le R$, there exists a unique solution $\mu \in C_T^0 \textsf{X}_{\phi ,\gamma }^{\sigma ,0,r,\frac{2q}{q-1}}$ to the Cauchy problem (3.1), with $T\ge C(|{\mathbb {M}}|R)^{-\frac{2s}{(2-\sigma )s-1}}$. Moreover,

3.10 $$\begin{aligned} \Vert \mu \Vert _{C_T^0\textsf{X}_{\phi ,\gamma }^{\sigma ,0,r,\frac{2q}{q-1}}} \le 2\Vert \mu ^0\Vert _{\textsf{X}_{\alpha ,\gamma }^{\sigma ,0,r,\frac{2q}{q-1}}}. \end{aligned}$$

Additionally, if $\Vert \mu _j^0\Vert _{\textsf{X}_{\alpha ,\gamma }^{\sigma ,0,r,\frac{2q}{q-1}}}\le R$, for $j\in \{1,2\}$, then

3.11 $$\begin{aligned} \Vert \mu _1-\mu _2\Vert _{C_T^0\textsf{X}_{\phi ,\gamma }^{\sigma ,0,r,\frac{2q}{q-1}}} \le 2\Vert \mu _1^0-\mu _2^0\Vert _{\textsf{X}_{\alpha ,\gamma }^{\sigma ,0,r,\frac{2q}{q-1}}}. \end{aligned}$$

### Remark 3.2

Compared to statement of Theorem 1.2, where the Sobolev indices $\sigma _r,\sigma _q$ can be arbitrarily large, Proposition 3.1 contains the restriction $\sigma <\frac{2s-1}{s}$ and the second Sobolev index is set to zero. These restrictions are temporary: we only need them to first obtain the existence of a solution. Using a monotonicity argument in the next section, which is in the spirit of persistence of regularity arguments, we then allow for larger values of $\sigma$.

### Remark 3.3

A lower bound for $r_0$ is explicitly worked out in the proof of Proposition 3.1. See condition (LWP2) below and the ensuing analysis.

### Remark 3.4

The solutions constructed by Proposition 3.1 do not a priori conserve mass.^4^ To see this, note that by using Eq. (3.1) and the fundamental theorem of calculus,

3.12 $$\begin{aligned} \frac{d}{dt}\int _{{\mathbb {R}}^d}\mu ^t(x)dx = -\frac{\nu ^2}{2}\int _{{\mathbb {R}}^d}(1+|\nabla |^s)^2\mu ^t(x)dx = -\frac{\nu ^2}{2}\int _{{\mathbb {R}}^d}\mu ^t(x)dx, \end{aligned}$$

where the ultimate equality follows from expanding the square and using the Fourier transform. Solving the ODE (3.12), we find

3.13 $$\begin{aligned} \int _{{\mathbb {R}}^d}\mu ^t(x)dx = e^{-\frac{\nu ^2 t}{2}}\int _{{\mathbb {R}}^d}\mu ^0(x)dx. \end{aligned}$$

So if $\mu ^0$ has zero mass, then $\mu ^t$ has zero mass for all times *t*. Otherwise, the magnitude of the mass is exponentially decreasing as $t\rightarrow \infty$. Recalling the mass/diffusion threshold for the PKS equation, this decreasing of the mass of our solutions may provide some intuition why global existence is ultimately possible.

### Gevrey embeddings

Before proceeding to the contraction mapping step, we record some elementary embeddings satisfied by the spaces ${\textsf{G}}_a^{\kappa ,r}$.

#### Lemma 3.5

If $a'\ge a\ge 0$ and $\kappa '\ge \kappa$, then

3.14 $$\begin{aligned} \Vert f\Vert _{{\textsf{G}}_{a}^{\kappa ,r}} \le e^{a-a'}\Vert f\Vert _{{\textsf{G}}_{a'}^{\kappa ',r}}. \end{aligned}$$

If $\kappa '\ge \kappa$ and $a'>a\ge 0$, then

3.15 $$\begin{aligned} \Vert f\Vert _{{\textsf{G}}_{a}^{\kappa ',r}} \le \frac{\lceil {\kappa '-\kappa }\rceil !}{(a'-a)^{\lceil {\kappa '-\kappa }\rceil }} \Vert f\Vert _{{\textsf{G}}_{a'}^{\kappa ,r}}. \end{aligned}$$

where $\lceil {\cdot }\rceil$ denotes the usual ceiling function.

#### Proof

First, observe that for any $a'\ge a\ge 0$,

3.16 $$\begin{aligned} \Vert f\Vert _{{\textsf{G}}_{a}^{\kappa ,r}} = \Vert e^{(a-a')A^{1/2}}e^{a' A^{1/2}}f\Vert _{{\hat{W}}^{\kappa s,r}} \le e^{a-a'}\Vert f\Vert _{{\textsf{G}}_{a'}^{\kappa ,r}}, \end{aligned}$$

since $e^{(a-a')(1+|\xi |^s)} \le e^{a-a'}$. Also, for any $\kappa '\ge \kappa$, we trivially have from $\Vert \cdot \Vert _{{\hat{W}}^{\kappa s, r}}\le \Vert \cdot \Vert _{{\hat{W}}^{\kappa ' s,r}}$ that

3.17 $$\begin{aligned} \Vert f\Vert _{{\textsf{G}}_{a}^{\kappa ,r}} \le \Vert f\Vert _{{\textsf{G}}_{a}^{\kappa ',r}}. \end{aligned}$$

Now for $\kappa '\ge \kappa$, we have

3.18 $$\begin{aligned} \Vert f\Vert _{{\textsf{G}}_{a}^{\kappa ',r}} = \Vert \langle \nabla \rangle ^{\kappa ' s}e^{a A^{1/2}}f\Vert _{{\hat{L}}^r} = \Vert \langle \nabla \rangle ^{(\kappa '-\kappa )s}e^{(a-a') A^{1/2}} \langle \nabla \rangle ^{\kappa s}e^{a' A^{1/2}}f\Vert _{{\hat{L}}^r}. \end{aligned}$$

Observe from the power series for $z\mapsto e^z$ that

3.19 $$\begin{aligned} \langle \xi \rangle ^{(\kappa '-\kappa ) s}e^{(a-a')(1+|\xi |^s)} \le (1+|\xi |^s)^{\kappa '-\kappa }e^{(a-a')(1+|\xi |^s)} \le \frac{\lceil {\kappa '-\kappa }\rceil !}{(a'-a)^{\lceil {\kappa '-\kappa }\rceil }}, \end{aligned}$$

where $\lceil {\cdot }\rceil$ is the usual ceiling function. Implicitly, we have used $\Vert \cdot \Vert _{\ell ^2}\le \Vert \cdot \Vert _{\ell ^s}$, since $s\le 2$. Therefore,

3.20 $$\begin{aligned} \Vert f\Vert _{{\textsf{G}}_{a}^{\kappa ',r}} \le \frac{\lceil {\kappa '-\kappa }\rceil !}{(a'-a)^{\lceil {\kappa '-\kappa }\rceil }} \Vert f\Vert _{{\textsf{G}}_{a'}^{\kappa ,r}}. \end{aligned}$$

$$\square $$

### Contraction mapping argument

Next, we define the map

3.21 $$\begin{aligned} \mu ^t\mapsto (\mathcal {T}\mu )^t :=e^{-\frac{t\nu ^2}{2}A}\mu ^0 - \int _0^t e^{-\frac{(t-\tau )\nu ^2}{2}A} B^\tau (\mu ^\tau ,\mu ^\tau )d\tau . \end{aligned}$$

We check that this map is well-defined on $C_T^0\textsf{X}_{\phi ,\gamma }^{\sigma ,0,r,\frac{2q}{q-1}}$ for $\phi ^t=\alpha +\beta t$, with $\alpha ,\beta ,\sigma ,r,q,\gamma >0$ satisfying the conditions in the statement of Proposition 3.1. To this end, we assume here and throughout this subsection that we have a realization of *W* belonging to $\Omega _{\alpha ,\beta ,\nu }$.

#### Lemma 3.6

If $\beta <\frac{\nu ^2}{2}$, then for any $1\le r\le \infty$, $0<s\le 1$, $\sigma \in {\mathbb {R}}$, and $\alpha >0$, it holds that

3.22 $$\begin{aligned} \Vert e^{-\frac{t\nu ^2}{2}A}\mu ^0\Vert _{{\textsf{G}}_{\phi ^t}^{\sigma ,r}} \le \Vert \mu ^0\Vert _{{\textsf{G}}_{\alpha }^{\sigma ,r}} \qquad \forall t\ge 0. \end{aligned}$$

#### Proof

It is straightforward from the definition of $\phi ^t$ that

3.23 $$\begin{aligned} \Vert e^{-\frac{t\nu ^2}{2}A}\mu ^0\Vert _{{\textsf{G}}_{\phi ^t}^{\sigma ,r}} = \Vert e^{t(\beta - \frac{\nu ^2}{2}A^{1/2})A^{1/2}}e^{\alpha A^{1/2}}\mu ^0\Vert _{{\hat{W}}^{\sigma s,r}} \end{aligned}$$

If $\beta <\frac{\nu ^2}{2}$, then since $(1+|\xi |^s)\ge 1$, the right-hand is $\le \Vert e^{\alpha A^{1/2}}\mu ^0\Vert _{{\hat{W}}^{\sigma s,r}} = \Vert \mu ^0\Vert _{{\textsf{G}}_{\alpha }^{\sigma ,r}}$. $\square$

Next, we observe from the bilinearity of *B* that

3.24 $$\begin{aligned} B(\mu _1,\mu _1) - B(\mu _2,\mu _2) = B(\mu _1-\mu _2,\mu _1) + B(\mu _2,\mu _1-\mu _2), \end{aligned}$$

and therefore

3.25 $$\begin{aligned} \left( \mathcal {T}(\mu _1) - \mathcal {T}(\mu _2)\right) ^t = -\int _0^t e^{-\frac{\nu ^2(t-\tau )}{2}A}\left( B^\tau (\mu _1^\tau -\mu _2^\tau , \mu _1^\tau ) + B^\tau (\mu _2^\tau ,\mu _1^\tau -\mu _2^\tau )\right) d\tau .\nonumber \\ \end{aligned}$$

#### Lemma 3.7

Let $d\ge 1$, $0<\gamma <d+1$, $\frac{1}{2}<s\le 1$. If $\gamma >1$, also assume that we are given $1\le q < \frac{d}{\gamma -1}$. Additionally, suppose that $\beta < \frac{\nu ^2}{2}$.

There exists an $r_0 \in [1,\infty ]$, depending on $d,\gamma ,s$, such that the following holds. For any $1\le r \le r_0$, there exists $\sigma _0 \in (0,\frac{2s-1}{s})$ depending on $d,\gamma ,s,r$, such that for any $\sigma \in (\sigma _0, \frac{2s-1}{s})$ with $1-\gamma \le \sigma s$, there exists a constant *C* depending only on $d,\gamma ,r,q,\sigma ,s,\beta ,\nu$, such that for any $T>0$,

3.26 $$\begin{aligned}&\left\| \int _0^t e^{-\frac{\nu ^2(t-\tau )}{2}A}B^\tau (\mu _1^\tau ,\mu _2^\tau )d\tau \right\| _{C_T^0{\textsf{G}}_{\phi }^{\sigma ,r}} \le C|{\mathbb {M}}|\left( T+T^{1-\frac{\sigma s+1}{2s}}\right) \Bigg (\Big (\Vert \mu _1\Vert _{C_T^0{\textsf{G}}_{\phi }^{0,r}}\Vert \mu _2 \Vert _{C_T^0{\textsf{G}}_{\phi }^{0,\frac{2q}{q-1}}} \nonumber \\&\quad + \Vert \mu _1\Vert _{C_T^0{\textsf{G}}_{\phi }^{0,\frac{2q}{q-1}}} \Vert \mu _2\Vert _{C_T^0{\textsf{G}}_{\phi }^{0,\frac{2q}{q-1}}}\Big )\textbf{1}_{\gamma >1} + \Vert \mu _1\Vert _{C_T^0{\textsf{G}}_{\phi }^{\sigma ,r}} \Vert \mu _2\Vert _{C_T^0{\textsf{G}}_{\phi }^{\sigma ,r}}\Bigg ). \end{aligned}$$

#### Remark 3.8

We give bounds on the size of the threshold $r_0$ from the statement of Lemma 3.7 during the proof of the lemma. We have omitted them from the statement in order to simplify the presentation. There is an extensive amount of algebra to determine the final conditions on the parameters, but if the reader is not interested in this optimization, they can just consider $r=s=1$, which is straightforward to check.

#### Proof of Lemma 3.7

We make the change of unknown $\mu _j^t:=e^{-\phi ^t A^{1/2}}\langle \nabla \rangle ^{-\sigma s} \rho _j^t$, so that

3.27 $$\begin{aligned} \Vert \rho _j^t\Vert _{{\hat{L}}^r} = \Vert \mu _j^t\Vert _{{\textsf{G}}_{\phi ^t}^{\sigma ,r}}. \end{aligned}$$

By Minkowski’s inequality, we see that

3.28 $$\begin{aligned} \Vert e^{\phi ^t A^{1/2}} \int _0^{t}e^{-\frac{\nu ^2(t-\tau )}{2}A}B^\tau (\mu _1^\tau ,\mu _2^\tau ) d\tau \Vert _{{\hat{W}}^{\sigma s,r}} \le \int _0^t \Vert e^{\phi ^t A^{1/2} - \frac{\nu ^2(t-\tau )}{2}A}B^\tau (\mu _1^\tau ,\mu _2^\tau )\Vert _{{\hat{W}}^{\sigma s,r}}d\tau , \end{aligned}$$

and by definition of the ${\hat{W}}^{\sigma s,r}$ norm, the preceding right-hand side equals

3.29 $$\begin{aligned}&\int _0^t \Bigg (\int _{{\mathbb {R}}^d} e^{r\phi ^t(1+|\xi |^s) - \nu ^2(t-\tau )(1+|\xi |^s)^2}\langle \xi \rangle ^{r\sigma s} \left| e^{-\nu W^\tau (1+|\xi |^s)}\int _{{\mathbb {R}}^d}\frac{(\xi \cdot {\mathbb {M}}\eta ){\hat{{\textsf{g}}}}(\eta )}{\langle \xi -\eta \rangle ^{\sigma s}\langle \eta \rangle ^{\sigma s}} \right. \nonumber \\&\quad \left. e^{-\phi ^\tau (2+|\xi -\eta |^s +|\eta |^s)}e^{\nu W^\tau (2+|\xi -\eta |^s+|\eta |^s)}{\hat{\rho }}_1^\tau (\xi -\eta ){\hat{\rho }}_2^\tau (\eta ) d\eta \right| ^r\Bigg )^{1/r}d\tau . \end{aligned}$$

Using $\phi ^t-\phi ^\tau = \beta (t-\tau )$, the preceding expression is controlled by

3.30 $$\begin{aligned}&\int _0^t\Bigg (\int _{{\mathbb {R}}^d}e^{r(t-\tau )(1+|\xi |^s)(\beta -\frac{\nu ^2}{2}(1+|\xi |^s))}\langle \xi \rangle ^{r\sigma s} \left| \int _{{\mathbb {R}}^d} e^{(\phi ^\tau -\nu W^{\tau })(|\xi |^s-|\xi -\eta |^s-|\eta |^s-1)} \right. \nonumber \\&\quad \left. \frac{|\xi \cdot {\mathbb {M}}\eta | |{\hat{{\textsf{g}}}}(\eta )|}{\langle \xi -\eta \rangle ^{\sigma s}\langle \eta \rangle ^{\sigma s}}\left| {\hat{\rho }}_1^\tau (\xi -\eta )\right| \left| {\hat{\rho }}_2^\tau (\eta )\right| d\eta \right| ^r d\xi \Bigg )^{1/r}d\tau . \end{aligned}$$

Since $0<s\le 1$, we have by the triangle inequality and $\Vert \cdot \Vert _{\ell ^1}\le \Vert \cdot \Vert _{\ell ^s}$ that $|\xi |^s - |\xi -\eta |^s-|\eta |^s\le 0$.^5^ Since $\phi ^\tau -\nu W^\tau \ge 0$ for all $0\le \tau \le t$ by assumption, it follows that

3.31 $$\begin{aligned} e^{(\phi ^\tau -\nu W^{\tau })(|\xi |^s-|\xi -\eta |^s-|\eta |^s-1)} \le 1. \end{aligned}$$

Now set $\delta :=\min \{\frac{1}{2}(\frac{\nu ^2}{2}-\beta ), \frac{\nu ^2}{4}\}$. By assumption that $\beta < \frac{\nu ^2}{2}$, we have

3.32 $$\begin{aligned} e^{r(t-\tau )(1+|\xi |^s)(\beta -\frac{\nu ^2}{2}(1+|\xi |^s))} \le e^{-r\delta (t-\tau )(1+|\xi |^s)^2}. \end{aligned}$$

With these observations, we reduce to estimate the expression

3.33 $$\begin{aligned} \int _0^t \Bigg (\int _{{\mathbb {R}}^d} e^{-r\delta (t-\tau )(1+|\xi |^s)^2}\langle \xi \rangle ^{r\sigma s}\left| \int _{{\mathbb {R}}^d}\frac{|\xi \cdot {\mathbb {M}}\eta | |{\hat{{\textsf{g}}}}(\eta )|}{\langle \xi -\eta \rangle ^{\sigma s}\langle \eta \rangle ^{\sigma s}}\left| {\hat{\rho }}_1^\tau (\xi -\eta )\right| \left| {\hat{\rho }}_2^\tau (\eta )\right| d\eta \right| ^r d\xi \Bigg )^{1/r} d\tau . \end{aligned}$$

To deal with the inhomogeneity of $(1+|\xi |^s)^2$, we split the integral with respect to $\xi$ into the low-frequency piece $|\xi |\le 1$ and the high-frequency piece $|\xi |>1$. At low frequency, we can crudely estimate everything directly to find

3.34 $$\begin{aligned}&\Bigg (\int _{|\xi |\le 1} e^{-r\delta (t-\tau )(1+|\xi |^s)^2}\left| \int _{{\mathbb {R}}^d}\frac{|\xi \cdot {\mathbb {M}}\eta | |{\hat{{\textsf{g}}}}(\eta )|}{\langle \xi -\eta \rangle ^{\sigma s}\langle \eta \rangle ^{\sigma s}}\left| {\hat{\rho }}_1^\tau (\xi -\eta )\right| \left| {\hat{\rho }}_2^\tau (\eta )\right| d\eta \right| ^r d\xi \Bigg )^{1/r} \nonumber \\&\quad \lesssim _\gamma |{\mathbb {M}}|\Bigg (\int _{|\xi |\le 1}\left| \int _{{\mathbb {R}}^d}\langle \xi -\eta \rangle ^{-\sigma s}|\eta |^{1-\gamma }\langle \eta \rangle ^{-\sigma s}\left| {\hat{\rho }}_1^\tau (\xi -\eta )\right| \left| {\hat{\rho }}_2^\tau (\eta )\right| d\eta \right| ^r d\xi \Bigg )^{1/r}. \end{aligned}$$

Above, we have used our assumption that $|\eta {\hat{{\textsf{g}}}}(\eta )|\lesssim _\gamma \eta ^{1-\gamma }$. If $1-\gamma \ge 0$, then $|\eta |^{1-\gamma }\langle \eta \rangle ^{-\sigma s} \le \langle \eta \rangle ^{1-\gamma -\sigma s}$. If $1-\gamma <0$, then we have to be careful about singularities at low frequency. More precisely, by Hölder’s inequality, we can control the $L_{\xi }^r$ norm by the $L_{\xi }^\infty$ norm. For $q(1-\gamma )>-d$, Hölder’s inequality gives

3.35 $$\begin{aligned}&\left| \int _{|\eta |\le 1}\langle \xi -\eta \rangle ^{-\sigma s}|\eta |^{1-\gamma }\langle \eta \rangle ^{-\sigma s} |{\hat{\rho }}_1^\tau (\xi -\eta )| |{\hat{\rho }}_2^\tau (\eta )|d\eta \right| \nonumber \\&\quad \le \Vert |\cdot |^{1-\gamma }1_{B(0,1)} \Vert _{L^q} \Vert \langle \xi -\cdot \rangle ^{-\sigma s}\langle \cdot \rangle ^{-\sigma s}{\hat{\rho }}_1^\tau (\xi -\cdot ) {\hat{\rho }}_2^\tau 1_{B(0,1)}\Vert _{L^{\frac{q}{q-1}}} \nonumber \\&\quad \lesssim _{d,\gamma ,q} \Vert {\hat{\rho }}_1^\tau \Vert _{{\hat{W}}^{-\sigma s,\frac{2q}{q-1}}} \Vert {\hat{\rho }}_2^\tau \Vert _{{\hat{W}}^{-\sigma s,\frac{2q}{q-1}}} \nonumber \\&\quad \lesssim \Vert e^{\phi ^\tau A^{1/2}}\mu _1^\tau \Vert _{{\hat{W}}^{0,\frac{2q}{q-1}}}\Vert e^{\phi ^\tau A^{1/2}}\mu _2^\tau \Vert _{{\hat{W}}^{0,\frac{2q}{q-1}}}. \end{aligned}$$

The remaining expression

3.36 $$\begin{aligned}&\Bigg (\int _{|\xi |\le 1} \left| \int _{|\eta |\ge 1}\langle \xi -\eta \rangle ^{-\sigma s}|\eta |^{1-\gamma }\langle \eta \rangle ^{-\sigma s}\left| {\hat{\rho }}_1^\tau (\xi -\eta )\right| \left| {\hat{\rho }}_2^\tau (\eta )\right| d\eta \right| ^r d\xi \Bigg )^{1/r} \end{aligned}$$

is handled by estimate (3.41) below.

At high frequency, we trivially have

3.37 $$\begin{aligned} e^{-r\delta (t-\tau )(1+|\xi |^s)^2}\le e^{-r\delta (t-\tau )|\xi |^{2s}}. \end{aligned}$$

Writing $|\xi | = (t-\tau )^{-\frac{1}{2s}}(t-\tau )^{\frac{1}{2s}}|\xi |$, it follows from the power series for $z\mapsto e^{z}$ that

3.38 $$\begin{aligned} e^{-r\delta (t-\tau )(1+|\xi |^s)^2}\langle \xi \rangle ^{r\sigma s}|\xi |^{r} \lesssim _\delta (t-\tau )^{-\frac{r(\sigma s+1)}{2s}}. \end{aligned}$$

Hence,

3.39 $$\begin{aligned}&\Bigg (\int _{|\xi |> 1} e^{-r\delta (t-\tau )(1+|\xi |^s)^2} \langle \xi \rangle ^{r\sigma s}\left| \int _{{\mathbb {R}}^d}\frac{|\xi \cdot {\mathbb {M}}\eta | |{\hat{{\textsf{g}}}}(\eta )|}{\langle \xi -\eta \rangle ^{\sigma s}\langle \eta \rangle ^{\sigma s}}\left| {\hat{\rho }}_1^\tau (\xi -\eta )\right| \left| {\hat{\rho }}_2^\tau (\eta )\right| d\eta \right| ^r d\xi \Bigg )^{1/r} \nonumber \\&\quad \lesssim _{\gamma ,\delta ,\sigma ,s} |{\mathbb {M}}|(t-\tau )^{-\frac{(\sigma s+1)}{2s}} \Bigg (\int _{|\xi |>1} \left| \int _{{\mathbb {R}}^d} \langle \xi -\eta \rangle ^{-\sigma s}|\eta |^{1-\gamma }\langle \eta \rangle ^{-\sigma s}\left| {\hat{\rho }}_1^\tau (\xi -\eta )\right| \left| {\hat{\rho }}_2^\tau (\eta )\right| d\eta \right| ^r d\xi \Bigg )^{1/r}. \end{aligned}$$

As before, we have to be careful about singularities in $\eta$ at low frequency if $1-\gamma < 0$. Observe from Young’s inequality that

3.40 $$\begin{aligned}&\Bigg (\int _{|\xi |>1} \left| \int _{|\eta |\le 1} \langle \xi -\eta \rangle ^{-\sigma s}|\eta |^{1-\gamma }\langle \eta \rangle ^{-\sigma s}\left| {\hat{\rho }}_1^\tau (\xi -\eta )\right| \left| {\hat{\rho }}_2^\tau (\eta )\right| d\eta \right| ^r d\xi \Bigg )^{1/r} \nonumber \\&\quad \le \Vert \langle \cdot \rangle ^{-\sigma s}{\hat{\rho }}_1^\tau \Vert _{L^r} \Vert |\cdot |^{1-\gamma }{\hat{\rho }}_2^\tau 1_{B(0,1)}\Vert _{L^1} \nonumber \\&\quad \lesssim _{d,\gamma ,\sigma ,s,q} \Vert e^{\phi ^\tau A^{1/2}} \mu _1^\tau \Vert _{{\hat{W}}^{0,r}} \Vert e^{\phi ^\tau A^{1/2}}\mu _2^\tau \Vert _{{\hat{W}}^{0,\frac{2q}{q-1}}}, \end{aligned}$$

for any $q<\frac{d}{\gamma -1}$. For $\eta$ at high frequency, we have by Young’s inequality followed by Lemma 2.1 that for any $1\le p\le r$,

3.41 $$\begin{aligned}&\Bigg (\int _{{\mathbb {R}}^d} \left| \int _{|\eta |\ge 1} \langle \xi -\eta \rangle ^{-\sigma s}\langle \eta \rangle ^{1-\gamma -\sigma s}\left| {\hat{\rho }}_1^\tau (\xi -\eta )\right| \left| {\hat{\rho }}_2^\tau (\eta )\right| d\eta \right| ^r d\xi \Bigg )^{1/r} \nonumber \\&\quad \le \Vert \langle \cdot \rangle ^{-\sigma s}{\hat{\rho }}_1^\tau \Vert _{L^p} \Vert \langle \cdot \rangle ^{1-\gamma -\sigma s} {\hat{\rho }}_2^\tau \Vert _{L^{\frac{rp}{(r+1)p-r}}} \nonumber \\&\quad \lesssim _{\sigma ,s,d,\gamma ,r,p} \Vert \rho _1^\tau \Vert _{{\hat{W}}^{-\sigma s, 1}} \Vert \rho _2^\tau \Vert _{{\hat{W}}^{1-\gamma -\sigma s, 1}}\textbf{1}_{r=1} + \Vert \rho _1^\tau \Vert _{{\hat{W}}^{(\frac{d(r-1)}{r}-\sigma s)+, r}} \Vert \rho _2^\tau \Vert _{{\hat{W}}^{1-\gamma -\sigma s, r}}\textbf{1}_{\begin{array}{c} p=1 \\ r>1 \end{array}} \nonumber \\&\qquad +\, \Vert \rho _1^\tau \Vert _{{\hat{W}}^{-\sigma s,r}} \Vert \rho _2^\tau \Vert _{{\hat{W}}^{(1-\gamma -\sigma s + \frac{d(r-1)}{r})+, r}}\textbf{1}_{\begin{array}{c} p=r \\ r>1 \end{array}}\nonumber \\&\qquad +\,\Vert \rho _1^\tau \Vert _{{\hat{W}}^{(\frac{d(r-p)}{rp}-\sigma s)+,r}} \Vert \rho _2^\tau \Vert _{{\hat{W}}^{(1-\gamma -\sigma s+\frac{d(p-1)}{p})+, r}}\textbf{1}_{\begin{array}{c} 1<p<r \\ r>1 \end{array}} \nonumber \\&\quad = \Vert e^{\phi ^\tau A^{1/2}}\mu _1^\tau \Vert _{{\hat{W}}^{0,1}}\Vert e^{\phi ^\tau A^{1/2}}\mu _2^\tau \Vert _{{\hat{W}}^{1-\gamma ,1}}\textbf{1}_{r=1} + \Vert e^{\phi ^\tau A^{1/2}}\mu _1^\tau \Vert _{{\hat{W}}^{\frac{d(r-1)}{r}+,r}}\Vert e^{\phi ^\tau A^{1/2}}\mu _2^\tau \Vert _{{\hat{W}}^{1-\gamma ,r}}\textbf{1}_{\begin{array}{c} p=1 \\ r>1 \end{array}} \nonumber \\&\qquad +\, \Vert e^{\phi ^\tau A^{1/2}}\mu _1^\tau \Vert _{{\hat{W}}^{0,r}}\Vert e^{ \phi ^\tau A^{1/2}}\mu _2^\tau \Vert _{{\hat{W}}^{(1-\gamma + \frac{d(r-1)}{r})+,r}}\textbf{1}_{\begin{array}{c} p=r \\ r>1 \end{array}}\nonumber \\&\qquad +\, \Vert e^{\phi ^\tau A^{1/2}}\mu _1^\tau \Vert _{{\hat{W}}^{(\frac{d(r-p)}{rp})+,r}} \Vert e^{\phi ^\tau A^{1/2}}\mu _2^\tau \Vert _{{\hat{W}}^{(1-\gamma +\frac{d(p-1)}{p})+, r}}\textbf{1}_{\begin{array}{c} 1<p<r \\ r>1 \end{array}} . \end{aligned}$$

In order to obtain estimates that close, we need the top Sobolev index appearing in (3.41) to be $\le \sigma s$. This leads us to the following conditions:

3.42 $$\begin{aligned} {\left\{ \begin{array}{ll} 1-\gamma \le \sigma s, &{} {r=1} \\ \frac{d(r-1)}{r}<\sigma s \ \text {and} \ 1-\gamma \le \sigma s, &{} {p=1 \ \text {and} \ r>1} \\ 1-\gamma +\frac{d(r-1)}{r}<\sigma s, &{} {p=r \ \text {and} \ r>1} \\ \frac{d(r-p)}{rp}<\sigma s \ \text {and} \ 1-\gamma +\frac{d(p-1)}{p}< \sigma s, &{} {1<p<r \ \text {and} \ r>1}. \end{array}\right. } \end{aligned}$$

Putting together the estimates (3.40) and (3.41), we have shown that

3.43 $$\begin{aligned}&\Bigg (\int _{|\xi |>1} \left| \int _{{\mathbb {R}}^d} \langle \xi -\eta \rangle ^{-\sigma s}|\eta |^{1-\gamma }\langle \eta \rangle ^{-\sigma s}\left| {\hat{\rho }}_1^\tau (\xi -\eta )\right| \left| {\hat{\rho }}_2^\tau (\eta )\right| d\eta \right| ^r d\xi \Bigg )^{1/r}\nonumber \\&\quad \lesssim _{d,s,\sigma ,\gamma ,r,q} \Vert e^{\phi ^\tau A^{1/2}} \mu _1^\tau \Vert _{{\hat{W}}^{0,r}}\Vert e^{\phi ^\tau A^{1/2}}\mu _2^\tau \Vert _{{\hat{W}}^{0,\frac{2q}{q-1}}}\textbf{1}_{\gamma >1} \nonumber \\&\qquad +\Vert e^{\phi ^\tau A^{1/2}} \mu _1^\tau \Vert _{{\hat{W}}^{\sigma s,r}} \Vert e^{\phi ^\tau A^{1/2}}\mu _2^\tau \Vert _{{\hat{W}}^{\sigma s,r}}. \end{aligned}$$

Combining the estimates (3.35) and (3.43), we have shown that (3.33) is $\lesssim _{d,\gamma ,s,\sigma ,r,q}$

3.44 $$\begin{aligned}&|{\mathbb {M}}|\int _0^t \left( 1+ C_\delta (t-\tau )^{-\frac{(\sigma s+1)}{2s}}\right) \Bigg (\Big (\Vert e^{\phi ^\tau A^{1/2}} \mu _1^\tau \Vert _{{\hat{W}}^{0,r}} \Vert e^{\phi ^\tau A^{1/2}}\mu _2^\tau \Vert _{{\hat{W}}^{0,\frac{2q}{q-1}}} \nonumber \\&\quad + \Vert e^{\phi ^\tau A^{1/2}} \mu _1^\tau \Vert _{{\hat{W}}^{0,\frac{2q}{q-1}}}\Vert e^{\phi ^\tau A^{1/2}} \mu _1^\tau \Vert _{{\hat{W}}^{0,\frac{2q}{q-1}}} \Big )\textbf{1}_{\gamma>1} +\Vert e^{\phi ^\tau A^{1/2}} \mu _1^\tau \Vert _{{\hat{W}}^{\sigma s,r}} \Vert e^{\phi ^\tau A^{1/2}}\mu _2^\tau \Vert _{{\hat{W}}^{\sigma s,r}}\Bigg )d\tau \nonumber \\&\quad \lesssim _{\sigma ,s} |{\mathbb {M}}|\left( t+C_\delta t^{1-\frac{(\sigma s+1)}{2s}}\right) \Bigg (\Big (\Vert \mu _1\Vert _{C_t^0{\textsf{G}}_{\phi }^{0,r}}\Vert \mu _2\Vert _{C_t^0{\textsf{G}}_{\phi }^{0,\frac{2q}{q-1}}} + \Vert \mu _1\Vert _{C_t^0{\textsf{G}}_{\phi }^{0,\frac{2q}{q-1}}} \Vert \mu _2\Vert _{C_t^0{\textsf{G}}_{\phi }^{0,\frac{2q}{q-1}}}\Big )\textbf{1}_{\gamma >1} \nonumber \\&\qquad + \Vert \mu _1\Vert _{C_t^0{\textsf{G}}_{\phi }^{\sigma ,r}} \Vert \mu _2\Vert _{C_t^0{\textsf{G}}_{\phi }^{\sigma ,r}}\Bigg ), \end{aligned}$$

assuming that $$\frac{(\sigma s+1)}{2s} <1$$.

In order to complete the proof of the lemma, it is important to list all the conditions we imposed on the parameters $d,\gamma ,\sigma ,s,r$ during the course of the above analysis:

1. $$0<s\le 1$$;
2.
  - $$r=1$$ and $$1-\gamma \le \sigma s$$,
  - or $r>1$ and $\frac{d(r-1)}{r}<\sigma s$ and $1-\gamma \le \sigma s$,
  - or $$r>1$$ and $$1-\gamma + \frac{d(r-1)}{r}<\sigma s$$,
  - or $r>1$ and $\exists p\in (1,r)$ such that $\frac{d(r-p)}{rp}<\sigma s$ and $1-\gamma +\frac{d(p-1)}{p}<\sigma s$;
3. $$\frac{(\sigma s+1)}{2s}<1$$

Condition (LWP3) means $\sigma < \frac{2s-1}{s}$ and since we require $\sigma >0$, we need $s>\frac{1}{2}$. Given any value of $0<\gamma < d+1$, (LWP2)a can be satisfied by choosing $r=1$ and $\sigma s\ge \min \{0,1-\gamma \}$. More generally, we can satisfy all three conditions by arguing as follows. Given $0<\gamma < d+1$ and $\frac{1}{2}<s\le 1$, condition (LWP3) implies that for any choice $r\ge 1$,

3.45 $$\begin{aligned} \sigma s < 2s-1. \end{aligned}$$

According to (LWP2)b, it is possible to find such a $\sigma \ge \frac{1-\gamma }{s}$ and $r>1$ if and only if

3.46 $$\begin{aligned} \frac{d(r-1)}{r}< 2s-1 \ \text {and} \ 1-\gamma< 2s-1 \quad \Longleftrightarrow \quad r< \frac{d}{d+1-2s} \ \text {and} \ \frac{2-\gamma }{2} < s . \end{aligned}$$

According to (LWP2)c, it is possible to find such a $\sigma >0$ and $r>1$ if and only if

3.47 $$\begin{aligned} 1-\gamma + \frac{d(r-1)}{r}< 2s-1 \quad \Longleftrightarrow \quad {\left\{ \begin{array}{ll} r<\frac{d}{d+2-\gamma -2s}, &{} {\gamma +2s\le d+2} \\ r\le \infty , &{}{\gamma +2s> d+2}.\quad \quad \end{array}\right. } \end{aligned}$$

According to (LWP2)d, it is possible to find such a $\sigma >0$ and $r>1$ if and only if for such choice of *r*, there exists $p\in (1,r)$ such that

3.48 $$\begin{aligned} \frac{d(r-p)}{rp}< 2s-1 \quad \text {and} \quad 1- \gamma + \frac{d(p-1)}{p} < 2s-1. \end{aligned}$$

Since the preceding constraint is equivalent to

3.49 $$\begin{aligned} \frac{d}{p}< \frac{d}{r}+2s-1 \quad \text {and} \quad d+2-\gamma -2s < \frac{d}{p}, \end{aligned}$$

such a *p* exists if and only if

3.50 $$\begin{aligned} d+2-\gamma -2s< \frac{d}{r}+2s-1 \quad \Longleftrightarrow \quad {\left\{ \begin{array}{ll} r<\frac{d}{d+3-\gamma -4s}, &{} \gamma +4s\le d+3 \\ r\le \infty , &{} \gamma +4s > d+3. \end{array}\right. } \end{aligned}$$

With this case analysis, the proof of Lemma 3.7 is now complete. $\square$

#### Lemma 3.9

Let $d\ge 1$, $1<\gamma <d+1$. Suppose that $\gamma ,r,s,\sigma$ satisfy the constraints of Lemma 3.7 and that

3.51 $$\begin{aligned} d+1-\gamma <\sigma s + \frac{d}{r}. \end{aligned}$$

Then for any $1\le q <\frac{d}{\gamma -1}$, there exists a constant $C>0$ depending on $d,\gamma ,r,q,s,\sigma ,\beta ,\nu$, such that for any $T>0$,

3.52 $$\begin{aligned}&\left\| \int _0^t e^{-\frac{\nu ^2(t-\tau )}{2}A}B^\tau (\mu _1^\tau ,\mu _2^\tau )d\tau \right\| _{C_T^0{\textsf{G}}_{\phi }^{0,\frac{2q}{q-1}}} \le C|{\mathbb {M}}|\left( T+T^{1-\frac{\sigma s+1}{2s}}\right) \nonumber \\&\quad \Bigg (\Vert \mu _1\Vert _{C_{T}^0{\textsf{G}}_{\phi }^{0,\frac{2q}{q-1}}}\Vert \mu _2\Vert _{C_{T}^0{\textsf{G}}_{\phi }^{0,\frac{2q}{q-1}}}+ \Vert \mu _1\Vert _{C_{T}^0{\textsf{G}}_{\phi }^{\sigma , r}} \Vert \mu _2\Vert _{C_{T}^0{\textsf{G}}_{\phi }^{0,\frac{2q}{q-1}}}\Bigg ). \end{aligned}$$

#### Proof

Set $q':=\frac{q}{q-1}$. The proof follows the exact same lines of Lemma 3.7 with *r* replaced by $2q'$. Using the estimates (3.35), (3.40), (3.41), we find that

3.53 $$\begin{aligned}&\left\| \int _0^t e^{-\frac{\nu ^2(t-\tau )}{2}A}B^\tau (\mu _1^\tau ,\mu _2^\tau )d\tau \right\| _{{\textsf{G}}_{\phi ^t}^{0,2q'}} \nonumber \\&\quad \lesssim _{d,s,q,\gamma ,\beta ,\nu ,p} |{\mathbb {M}}|\int _0^t \left( 1+ (t-\tau )^{-\frac{\sigma s+1}{2s}}\right) \Bigg (\Vert e^{\phi ^\tau A^{1/2}}\mu _1^\tau \Vert _{{\hat{W}}^{0,2q'}}\Vert e^{\phi ^\tau A^{1/2}}\mu _2^\tau \Vert _{{\hat{W}}^{0,2q'}}\nonumber \\&\qquad +\Vert e^{\phi ^\tau A^{1/2}}\mu _1^\tau \Vert _{{\hat{W}}^{0,p}} \Vert e^{\phi ^\tau A^{1/2}} \mu _2^\tau \Vert _{{\hat{W}}^{1-\gamma ,\frac{2q'p}{(2q'+1)p-2q'}}}\Bigg )d\tau \end{aligned}$$

for any choice $1\le p\le 2q'$. We want all the norms appearing in the right-hand side to be controlled by ${\hat{W}}^{0,2q'}$ and ${\hat{W}}^{\sigma s, r}$. Using Lemma 2.1, we see that we need to choose $p\le r$ so that

3.54 $$\begin{aligned} \frac{d}{p} - \frac{d}{r} < \sigma s \Longleftrightarrow {\sigma s + \frac{d}{r}} > \frac{d}{p}. \end{aligned}$$

For any choice of *p*, we have

3.55 $$\begin{aligned} \frac{2q'p}{(2q'+1)p-2q'} = 2q'\left( \frac{p}{p + 2q'(p-1)}\right) \le 2q', \end{aligned}$$

since $p\ge 1$. In order for

3.56 $$\begin{aligned} \Vert e^{\phi ^\tau A^{1/2}} \mu _2^\tau \Vert _{{\hat{W}}^{1-\gamma ,\frac{2q'p}{(2q'+1)p-2q'}}} \lesssim \Vert e^{\phi ^\tau A^{1/2}} \mu _2^\tau \Vert _{{\hat{W}}^{0,2q'}}, \end{aligned}$$

another use of Lemma 2.1 tells us that we need

3.57 $$\begin{aligned} 1-\gamma + d\left( \frac{(2q' + 1)p - 2q'}{2q' p} - \frac{1}{2q'}\right) = 1-\gamma +\frac{d(p-1)}{p}< 0 \Longleftrightarrow d+1-\gamma < \frac{d}{p}. \end{aligned}$$

Since we also needed $\sigma s+\frac{d}{r}>\frac{d}{p}$, the existence of a *p* satisfying both the upper and lower bounds is true if and only if

3.58 $$\begin{aligned} d+1-\gamma< \sigma s + \frac{d}{r} \Longleftrightarrow {\left\{ \begin{array}{ll} r < \frac{d}{d+1-\gamma -\sigma s}, &{} \gamma +\sigma s \le d+1 \\ r\le \infty , &{} \gamma +\sigma s>d+1.\end{array}\right. } \end{aligned}$$

Note that if $\gamma \ge 1$, then for any $\sigma s>0$, we have $\frac{d}{d+1-\gamma -\sigma s} > 1$.

Under the constraints of the preceding paragraph, the right-hand side of (3.53) is $\lesssim _{d,\gamma ,r,q,\sigma , s}$

3.59 $$\begin{aligned}&|{\mathbb {M}}|\int _0^t \left( 1+(t-\tau )^{-\frac{\sigma s+1}{2s}}\right) \Bigg (\Vert e^{\phi ^\tau A^{1/2}}\mu _1^\tau \Vert _{{\hat{W}}^{0,2q'}}\Vert e^{\phi ^\tau A^{1/2}}\mu _2^\tau \Vert _{{\hat{W}}^{0,2q'}} \nonumber \\&\quad + \Vert e^{\phi ^\tau A^{1/2}}\mu _1^\tau \Vert _{{\hat{W}}^{\sigma s, r}} \Vert e^{\phi ^\tau A^{1/2}}\mu _2^\tau \Vert _{{\hat{W}}^{0, 2q'}} \Bigg )d\tau . \end{aligned}$$

Taking the supremum over $\tau \in [0,T]$ in the right-hand side and using fundamental theorem of calculus leads to the desired conclusion. $\square$

An immediate corollary of Lemmas 3.7 and 3.9 is the following estimate for the Duhamel term.

#### Corollary 3.10

Under the assumptions of Lemmas 3.7 and 3.9, there exists a constant $C>0$ depending on $d,\gamma ,r,q,\sigma , s, \beta ,\nu$, such that for any $T>0$ and $\mu _1,\mu _2 \in C_T^0\textsf{X}_{\phi ,\gamma }^{\sigma ,0,r,\frac{2q}{q-1}}$,

3.60 $$\begin{aligned}&\left\| \int _0^t e^{-\frac{\nu ^2(t-\tau )}{2}A}B^\tau (\mu _1^\tau ,\mu _2^\tau )d\tau \right\| _{C_T^0\textsf{X}_{\phi ,\gamma }^{\sigma ,0,r,\frac{2q}{q-1}}} \nonumber \\&\quad \le C|{\mathbb {M}}|\left( T+T^{1-\frac{\sigma s+1}{2s}}\right) \Bigg (\Vert \mu _1\Vert _{C_T^0\textsf{X}_{\phi ,\gamma }^{\sigma ,0,r,\frac{2q}{q-1}}}\Vert \mu _2\Vert _{C_T^0\textsf{X}_{\phi ,\gamma }^{\sigma ,0,r, \frac{2q}{q-1}}}\Bigg ). \end{aligned}$$

#### Proof of Proposition 3.1

Putting together the estimates of Lemma 3.6 and Corollary 3.10, we have shown that there exists a constant $C>0$ depending on $d,\gamma ,r,q,\sigma ,s,\beta ,\nu$, such that

3.61 $$\begin{aligned} \Vert \mathcal {T}(\mu )\Vert _{C_T^0\textsf{X}_{\phi ,\gamma }^{\sigma ,0,r,\frac{2q}{q-1}}} \le \Vert \mu ^0\Vert _{\textsf{X}_{\alpha ,\gamma }^{\sigma ,0,r,\frac{2q}{q-1}}} + C|{\mathbb {M}}|\left( T+T^{1-\frac{(\sigma s+1)}{2s}}\right) \Vert \mu \Vert _{C_T^0\textsf{X}_{\phi ,\gamma }^{\sigma ,0,r,\frac{2q}{q-1}}}^2 \end{aligned}$$

and

3.62 $$\begin{aligned}&\Vert \mathcal {T}(\mu _1)-\mathcal {T}(\mu _2)\Vert _{C_T^0\textsf{X}_{\phi ,\gamma }^{\sigma ,0,r,\frac{2q}{q-1}}} \le C|{\mathbb {M}}|\left( T +T^{1-\frac{(\sigma s+1)}{2s}}\right) \Vert \mu _1-\mu _2\Vert _{C_T^0\textsf{X}_{\phi ,\gamma }^{\sigma ,0,r,\frac{2q}{q-1}}}\nonumber \\&\quad \times \left( \Vert \mu _1\Vert _{C_T^0\textsf{X}_{\phi ,\gamma }^{\sigma ,0,r,\frac{2q}{q-1}}}+\Vert \mu _2\Vert _{C_T^0\textsf{X}_{\phi ,\gamma }^{\sigma ,0,r,\frac{2q}{q-1}}}\right) . \end{aligned}$$

We now want to show that for any appropriate choice of *T*, the map $\mathcal {T}$ is a contraction on the closed ball $B_{R}(0)$ of radius $R>2\Vert \mu ^0\Vert _{\textsf{X}_{\alpha ,\gamma }^{\sigma ,0,r,\frac{2q}{q-1}}}$ centered at the origin in the space $C_T^0\textsf{X}_{\phi ,\gamma }^{\sigma ,0,r,\frac{2q}{q-1}}$. Indeed, from the estimates (3.61) and (3.62), we see that if

3.63 $$\begin{aligned} C|{\mathbb {M}}|R\left( T+T^{1-\frac{\sigma s+1}{2s}}\right) {<} \frac{1}{2}, \end{aligned}$$

then $\mathcal {T}$ is a contraction on $B_R(0)$. So by the contraction mapping theorem, there exists a unique fixed point $\mu =\mathcal {T}(\mu ) \in C_T^0\textsf{X}_{\phi ,\gamma }^{\sigma ,0,r,\frac{2q}{q-1}}$. We note that $T\ge C'(|{\mathbb {M}}|R)^{-\frac{2s}{2s-\sigma s-1}}$, where $C'>0$ is a possibly different constant than *C* but depending on the same parameters.

The preceding result shows local existence and uniqueness of solutions to the Cauchy problem (3.1). To complete the proof of Proposition 3.1, we now prove continuous dependence on the initial data. For $j=1,2$, let $\mu _j$ be a solution in $C_{T_j}^{0}\textsf{X}_{\phi ,\gamma }^{\sigma ,0,r,\frac{2q}{q-1}}$ to (3.1) with initial datum $\mu _j^0$, such that $\Vert \mu _j^0\Vert _{\textsf{X}_{\alpha ,\gamma }^{\sigma ,0,r,\frac{2q}{q-1}}}\le R$. Then there exists a $T \gtrsim _{d,\gamma ,r,q,\sigma ,s,\beta ,\nu } (|{\mathbb {M}}|R)^{-\frac{2s}{2s-\sigma s-1}}$ such that $\mu _1,\mu _2$ are defined on [0, *T*]. From the mild formulation (3.3), the triangle inequality, Lemma 3.6, and Corollary 3.10, we see that

3.64 $$\begin{aligned}&\Vert \mu _1-\mu _2\Vert _{C_T^0\textsf{X}_{\phi ,\gamma }^{\sigma ,0,r,\frac{2q}{q-1}}} \le \Vert \mu _1^0-\mu _2^0 \Vert _{\textsf{X}_{\alpha ,\gamma }^{\sigma ,0,r,\frac{2q}{q-1}}} \nonumber \\&\quad + C|{\mathbb {M}}|\left( T+T^{1-\frac{\sigma s+1}{2s}}\right) \Vert \mu _1-\mu _2\Vert _{C_T^0\textsf{X}_{\phi ,\gamma }^{\sigma ,0,r,\frac{2q}{q-1}}}\left( \Vert \mu _1\Vert _{C_T^0 \textsf{X}_{\phi ,\gamma }^{\sigma ,0,r,\frac{2q}{q-1}}} + \Vert \mu _2\Vert _{C_T^0\textsf{X}_{\phi ,\gamma }^{\sigma ,0,r,\frac{2q}{q-1}}}\right) . \end{aligned}$$

Taking *T* smaller if necessary while still preserving $T\gtrsim _{d,\gamma ,r,q,s,\sigma ,\beta ,\nu } (|{\mathbb {M}}|R)^{-\frac{2s}{2s-\sigma s-1}}$, we may assume that $2C|{\mathbb {M}}|(T+T^{1-\frac{\sigma s+1}{2}})R \le \frac{1}{4}$. Bounding each $\Vert \mu _j\Vert _{C_T^0\textsf{X}_{\phi ,\gamma }^{\sigma ,0,r,\frac{2q}{q-1}}}$ by *R* in the last factor, it then follows from (3.64) that

3.65 $$\begin{aligned} \Vert \mu _1-\mu _2\Vert _{C_T^0\textsf{X}_{\phi ,\gamma }^{\sigma ,0,r,\frac{2q}{q-1}}}\le 2\Vert \mu _1^0-\mu _2^0\Vert _{\textsf{X}_{\alpha ,\gamma }^{\sigma ,0,r,\frac{2q}{q-1}}}. \end{aligned}$$

With this last estimate, the proof of Proposition 3.1 is complete. $\square$

## Global existence

We now show that with quantifiable high probability, there exists a global solution $\mu \in C_{\infty }^0\textsf{X}_{\phi ,\gamma }^{\sigma _r,\sigma _q,r,\frac{2q}{q-1}}$ to the Cauchy problem (3.1), provided $\sigma _r,\sigma _q,r,q$ are appropriately chosen. Moreover, the function

4.1 $$\begin{aligned} t \mapsto \Vert \mu ^t\Vert _{\textsf{X}_{\phi ^t,\gamma }^{\sigma _r,\sigma _q,r,q}} \end{aligned}$$

is strictly decreasing on $[0,\infty )$, provided that $\Vert \mu ^0\Vert _{\textsf{X}_{\alpha ,\gamma }^{\sigma _r,\sigma _q,r,q}}$ is sufficiently small. This then proves Theorem 1.2.

### Monotonicity of Gevrey norm

The goal of this subsection is to show the following. Suppose we have a solution $\mu \in C_T^0\textsf{X}_{\phi ,\gamma }^{\kappa _r,\kappa _q, r,\frac{2q}{q-1}}$ to (3.1), where $\phi ^t:=\alpha +\beta t$, such that $\mu$ also belongs to $C_T^0\textsf{X}_{\phi ,\gamma }^{\kappa _r',\kappa _q',r,\frac{2q}{q-1}}$, for sufficiently larger $\kappa _r'>\kappa _r$ and $\kappa _q'>\kappa _q$, and such that $\Vert \mu ^0\Vert _{\textsf{X}_{\alpha ,\gamma }^{\kappa _r,\kappa _q,r,\frac{2q}{q-1}}}$ is sufficiently small depending on $d,\gamma , r,q,\kappa _r,\kappa _q,s,\beta ,\nu ,|{\mathbb {M}}|$. If $\kappa _r,\kappa _q$ are sufficiently large depending on $d,s,\gamma$, then the quantity $\Vert \mu ^t\Vert _{\textsf{X}_{\phi ^t,\gamma }^{\kappa _r,\kappa _q, r,\frac{2q}{q-1}}}$ must be strictly decreasing on the interval [0, *T*]. In other words, the Gevrey norm of $\mu ^t$ is strictly decreasing on an interval, provided that we know a Gevrey norm with higher Sobolev index (but the same Gevrey index) remains finite on the same interval.

#### Proposition 4.1

Let $d\ge 1$, $0<\gamma <d+1$, $1\le r\le \infty$, $\frac{1}{2}<s\le 1$. If $\gamma >1$, then also suppose we are given $1<q<\frac{d}{\gamma -1}$. Given $\alpha ,\beta >0$, set $\phi ^t:=\alpha +\beta t$. Assume that *W* is a realization from $\Omega _{\alpha ,\beta ,\nu }$.

If $\gamma \le 1$, then there is a threshold $\kappa _{0,r}\in {\mathbb {R}}$ depending on $r,d,s,\gamma$, such that for any $\kappa _r>\kappa _{0,r}$, the following holds. There is a constant $C_r>0$, depending only on $d,\gamma ,r,s,\kappa _r$, such that if $\mu \in C_T^0{\textsf{G}}_{\phi }^{\kappa _r+\frac{2}{r},r}$ is a nonzero solution to (3.1), for some $T>0$, satisfying

4.2 $$\begin{aligned} \Vert \mu ^0\Vert _{{\textsf{G}}_{\alpha }^{\kappa _r,r}} < \frac{\nu ^2-2\beta }{C_r|{\mathbb {M}}|}, \end{aligned}$$

then

4.3 $$\begin{aligned} \Vert \mu ^t\Vert _{{\textsf{G}}_{\phi ^t}^{\kappa _r,r}}< \Vert \mu ^{t'}\Vert _{{\textsf{G}}_{\phi ^{t'}}^{\kappa _r,r}} \qquad \forall 0\le t' < t \le T. \end{aligned}$$

If $\gamma >1$, then there is a threshold $\kappa _{0,q}\in {\mathbb {R}}$ depending on $q,d,s,\gamma$, such that for any $\kappa _q>\kappa _{0,q}$, the following holds. There is a constant $C_q>0$, depending only on $d,\gamma ,q,s,\kappa _q$, such that if $\mu \in C_T^0{\textsf{G}}_{\phi }^{\kappa _q+\frac{q-1}{q}, \frac{2q}{q-1}}$ is a nonzero solution to (3.1), for some $T>0$, satisfying

4.4 $$\begin{aligned} \Vert \mu ^0\Vert _{{\textsf{G}}_{\alpha }^{\kappa _q,\frac{2q}{q-1}}} < \frac{\nu ^2-2\beta }{C_q|{\mathbb {M}}|}, \end{aligned}$$

then

4.5 $$\begin{aligned} \Vert \mu ^t\Vert _{{\textsf{G}}_{\phi ^t}^{\kappa _q,\frac{2q}{q-1}}}< \Vert \mu ^{t'}\Vert _{{\textsf{G}}_{\phi ^{t'}}^{\kappa _q,\frac{2q}{q-1}}} \qquad \forall 0\le t' < t \le T. \end{aligned}$$

Furthermore, if $\mu \in C_T^0\textsf{X}_{\phi ,\gamma }^{\kappa _r+\frac{2}{r},\kappa _q+\frac{q-1}{q},r,\frac{2q}{q-1}}$ is a nonzero solution, where $\kappa _r>\kappa _{0,r}$, such that

4.6 $$\begin{aligned} \Vert \mu ^0\Vert _{\textsf{X}_{\alpha ,\gamma }^{\kappa _r,\kappa _q,r,\frac{2q}{q-1}}} < \frac{\nu ^2-2\beta }{|{\mathbb {M}}|\max \{C_r,C_q\}}, \end{aligned}$$

then (4.3) also holds.

#### Remark 4.2

Bounds for the thresholds $\kappa _{0,r},\kappa _{0,q}$ are explicitly worked out in the proof of Proposition 4.1. See the conditions (U1),(U2) and (L1),(L2) below together with their respectively ensuing analysis.

The proof of Proposition 4.1 consists of several lemmas. To begin, we observe from the chain rule and using Eq. (3.1) (there is an approximation step we omit),

4.7 $$\begin{aligned}&\frac{d}{dt} |e^{\phi ^t(1+|\xi |^s)}{\hat{\mu }}^t(\xi )| = \Re \Bigg (|e^{\phi ^t(1+|\xi |^s)}{\hat{\mu }}^t(\xi )|^{-1} \overline{e^{\phi ^t (1+|\xi |^s)} {\hat{\mu }}^t(\xi )}\Bigg (\beta (1+|\xi |^s)e^{\phi ^t (1+|\xi |^s)} {\hat{\mu }}^t(\xi )\nonumber \\&\quad - e^{\phi ^t(1+|\xi |^s)}{\mathcal {F}}(B^t(\mu ^t,\mu ^t))(\xi ) - \frac{\nu ^2}{2}(1+|\xi |^s)^2 e^{\phi ^t(1+|\xi |^s)}{\hat{\mu }}^t \Bigg ). \end{aligned}$$

Replacing the first and second terms by their magnitudes, we see that the right-hand side is $\le$

4.8 $$\begin{aligned} |e^{\phi ^t(1+|\xi |^s)}{\hat{\mu }}^t(\xi )|\Bigg (\beta (1+|\xi |^s) - \frac{\nu ^2}{2}(1+|\xi |^s)^2\Bigg ) + \left| e^{\phi ^t(1+|\xi |^s)}{\mathcal {F}}(B^t(\mu ^t,\mu ^t))(\xi )\right| .\nonumber \\ \end{aligned}$$

Using the elementary inequality (remember that $s\le 1$)

4.9 $$\begin{aligned} \langle \xi \rangle \le (1+|\xi |^s)^{\frac{1}{s}} \le 2^{\frac{2-s}{2s}} \langle \xi \rangle \end{aligned}$$

together with our assumption that $\beta <\frac{\nu ^2}{2}$, we arrive at the inequality

4.10 $$\begin{aligned} \frac{d}{dt}|e^{\phi ^t(1+|\xi |^s)}{\hat{\mu }}^t(\xi )| \le -\langle \xi \rangle ^{2s} |e^{\phi ^t(1+|\xi |^s)}{\hat{\mu }}^t(\xi )|\left( \frac{\nu ^2}{2}-\beta \right) + \left| e^{\phi ^t(1+|\xi |^s)}{\mathcal {F}}(B^t(\mu ^t,\mu ^t))(\xi )\right| . \end{aligned}$$

It now follows from this identity, the chain rule, and differentiating inside the integral that for any $1\le r<\infty$,

4.11 $$\begin{aligned}&\frac{1}{r}\frac{d}{dt} \Vert e^{\phi ^t A^{1/2}}\mu ^t\Vert _{{\hat{W}}^{\sigma s,r}}^r \le -\left( \frac{\nu ^2}{2} -\beta \right) \int _{{\mathbb {R}}^d} \left| e^{\phi ^t (1+|\xi |^s)}\langle \xi \rangle ^{(\sigma +\frac{2}{r})s} {\hat{\mu }}^t(\xi ) \right| ^{r}d\xi \nonumber \\&\quad + \int _{{\mathbb {R}}^d} \left| e^{\phi ^t (1+|\xi |^s)}\langle \xi \rangle ^{\sigma s} {\hat{\mu }}^t(\xi ) \right| ^{r-1}\langle \xi \rangle ^{\sigma s} e^{\phi ^t(1+|\xi |^s)}|{\mathcal {F}}(B^t(\mu ^t,\mu ^t))(\xi )|d\xi . \end{aligned}$$

We need to show that the second term in (4.11) is not so large that it cannot be absorbed by the first term, which is negative. This is a problem in Fourier analysis, which we address with the next two lemmas.

#### Lemma 4.3

For any $t>0$ with $\phi ^t - \nu W^t\ge 0$, it holds for any test functions *f*, *g* that

4.12 $$\begin{aligned}&|e^{\phi ^t(1+|\xi |^s)}{\mathcal {F}}(B^t(f,g))(\xi )|\nonumber \\&\quad \lesssim _\gamma |{\mathbb {M}}|\int _{{\mathbb {R}}^d}|\xi | |\eta |^{1-\gamma } \left| e^{\phi ^t(1+|\xi -\eta |^s)}{\hat{f}}(\xi -\eta ) e^{\phi ^t(1+|\eta |^s)}{\hat{g}}(\eta )\right| d\eta . \end{aligned}$$

#### Proof

We observe from the definition (3.2) of $B^t$ that for any test functions *f*, *g*,

4.13 $$\begin{aligned}&e^{\phi ^t(1+|\xi |^s)}{\mathcal {F}}(B^t(f,g))(\xi ) \nonumber \\&\quad = \int _{{\mathbb {R}}^d}e^{\phi ^t(1+|\xi |^s) - \nu W^t(|\xi |^s - |\eta |^s - |\xi -\eta |^s-1)} \left( \xi \cdot {\mathbb {M}}\eta \right) {\hat{{\textsf{g}}}}(\eta ) {\hat{f}}(\xi -\eta ){\hat{g}}(\eta ) d\eta . \end{aligned}$$

Writing $1=e^{\phi ^t(1+|\eta |^s)}e^{-\phi ^t(1+|\eta |^s)} = e^{\phi ^t(1+|\xi -\eta |^s)}e^{-\phi ^t(1+|\xi -\eta |^s)}$, we see that the magnitude of the preceding right-hand side is controlled by

4.14 $$\begin{aligned}&\left| \int _{{\mathbb {R}}^d} e^{(\phi ^t-\nu W^t)(|\xi |^s-|\eta |^s-|\xi -\eta |^s-1)}\left( \xi \cdot {\mathbb {M}}\eta \right) {\hat{{\textsf{g}}}}(\eta ) e^{\phi ^t(1+|\xi -\eta |^s)}{\hat{f}}(\xi -\eta ) e^{\phi ^t(1+|\eta |^s)}{\hat{g}}(\eta )d\eta \right| \nonumber \\&\quad \lesssim _\gamma |{\mathbb {M}}|\int _{{\mathbb {R}}^d} e^{(\phi ^t-\nu W^t)(|\xi |^s-|\eta |^s-|\xi -\eta |^s-1)} |\xi | |\eta |^{1-\gamma }\left| e^{\phi ^t(1+|\xi -\eta |^s)}{\hat{f}}(\xi -\eta ) e^{\phi ^t(1+|\eta |^s)}{\hat{g}}(\eta )\right| d\eta , \end{aligned}$$

where we have used our assumption that $|\eta {\hat{{\textsf{g}}}}(\eta )|\lesssim _{\gamma } |\eta |^{1-\gamma }$. Since $\phi ^t-\nu W^t\ge 0$ by assumption and $|\xi |^s-|\eta |^s-|\xi -\eta |^s-1\le 0$ by $\Vert \cdot \Vert _{\ell ^1} \le \Vert \cdot \Vert _{\ell ^s}$ (recall $s\le 1$), the desired inequality now follows. $\square$

Next, we observe that by applying Lemma 4.3 to the second term in the right-hand side of (4.11), we need to estimate expressions of the form

4.15 $$\begin{aligned} \int _{{\mathbb {R}}^d} \left| e^{\phi ^t (1+|\xi |^s)}\langle \xi \rangle ^{\kappa s} {\hat{h}}(\xi ) \right| ^{r-1}\langle \xi \rangle ^{\kappa s+1}\int _{{\mathbb {R}}^d}|\eta |^{1-\gamma } \left| e^{\phi ^t(1+|\xi -\eta |^s)}{\hat{f}}(\xi -\eta ) e^{\phi ^t(1+|\eta |^s)}{\hat{g}}(\eta )\right| d\eta d\xi , \end{aligned}$$

where *f*, *g*, *h* are test functions. We take care of such expressions with the next lemma.

#### Lemma 4.4

Let $d\ge 1$, $0<\gamma <d+1$, $1\le r\le \infty$, $\frac{1}{2}< s\le 1$. If $\gamma >1$, also assume that $1\le q<\frac{d}{\gamma -1}$. Then there exists a threshold $\kappa _0$ depending on $d,\gamma ,r,s$, such that for any $\kappa >\kappa _0$, there exists a constant $C>0$ depending on $d,\gamma ,r,s,q,\kappa$ so that

4.16 $$\begin{aligned}&\int _{{\mathbb {R}}^d} \left| e^{\phi ^t (1+|\xi |^s)}\langle \xi \rangle ^{\kappa s} {\hat{h}}(\xi ) \right| ^{r-1}\langle \xi \rangle ^{\kappa s+1}\int _{{\mathbb {R}}^d}|\eta |^{1-\gamma } \left| e^{\phi ^t(1+|\xi -\eta |^s)}{\hat{f}}(\xi -\eta ) e^{\phi ^t(1+|\eta |^s)}{\hat{g}}(\eta )\right| d\eta d\xi \nonumber \\&\quad \le C\Vert e^{\phi ^t A^{1/2}} h\Vert _{{\hat{W}}^{(\kappa +\frac{2}{r})s,r}}^{r-1}\Bigg (\Vert e^{\phi ^t A^{1/2}}f\Vert _{{\hat{W}}^{(\kappa -\frac{2(r-1)}{r})s+1, r}} \Vert e^{\phi ^t A^{1/2}} g\Vert _{{\hat{W}}^{1-\gamma ,\frac{2q}{q-1}}}\textbf{1}_{\gamma >1} \nonumber \\&\qquad + \Vert e^{\phi ^t A^{1/2}}f\Vert _{{\hat{W}}^{(\kappa + \frac{2}{r})s,r}} \Vert e^{\phi ^t A^{1/2}}g\Vert _{{\hat{W}}^{\kappa s,r}} + \Vert e^{\phi ^t A^{1/2}}f\Vert _{{\hat{W}}^{\kappa s,r}}\Vert e^{\phi ^t A^{1/2}}g\Vert _{{\hat{W}}^{(\kappa + \frac{2}{r})s,r}}\Bigg ). \end{aligned}$$

#### Proof

Writing $\langle \xi \rangle ^{\kappa s+1} = \langle \xi \rangle ^{\kappa s+1-\frac{2s(r-1)}{r}}\langle \xi \rangle ^{\frac{2s(r-1)}{r}}$ and applying Hölder’s inequality with conjugate exponents $\frac{r}{r-1}$ and *r*, we find that the left-hand side of (4.16) is $\le$

4.17 $$\begin{aligned}&\Vert e^{\phi ^t A^{1/2}}\langle \nabla \rangle ^{(\kappa + \frac{2}{r})s} h\Vert _{{\hat{L}}^r}^{r-1}\Bigg (\int _{{\mathbb {R}}^d} \langle \xi \rangle ^{(r\kappa - 2(r-1))s+r} \left| \int _{{\mathbb {R}}^d}|\eta |^{1-\gamma } \right. \nonumber \\&\quad \left. \left| e^{\phi ^t(1+|\xi -\eta |^s)}{\hat{f}}(\xi -\eta ) e^{\phi ^t(1+|\eta |^s)}{\hat{g}}(\eta )\right| d\eta \right| ^r d\xi \Bigg )^{1/r}, \end{aligned}$$

with obvious modification if $r=\infty$. We need to estimate the second factor. We first address the singularity in $\eta$ at low frequency if $1-\gamma <0$. Observe that by separately considering the regions $|\xi | \le 2|\eta |$ and $|\xi |>2|\eta |$, it follows from Young’s inequality that

4.18 $$\begin{aligned}&\Bigg (\int _{{\mathbb {R}}^d} \langle \xi \rangle ^{(r\kappa - 2(r-1))s+r} \left| \int _{|\eta |\le 1}|\eta |^{1-\gamma }\left| \left( e^{\phi ^t(1+|\xi -\eta |^s)}{\hat{f}}(\xi -\eta )\right) \left( e^{\phi ^t(1+|\eta |^s)}{\hat{g}}(\eta )\right) \right| d\eta \right| ^r d\xi \Bigg )^{1/r} \nonumber \\&\quad \lesssim _{r,\kappa ,s,\gamma ,d} \Vert \langle \cdot \rangle ^{(\kappa -\frac{2(r-1)}{r})s+1} e^{\phi ^t(1+|\cdot |^s)}{\hat{f}}\Vert _{L^r} \Vert |\cdot |^{1-\gamma }e^{\phi ^t(1+|\cdot |^s)} {\hat{g}}1_{B(0,1)}\Vert _{L^1} \nonumber \\&\quad \lesssim \Vert e^{\phi ^t A^{1/2}}f\Vert _{{\hat{W}}^{(\kappa -\frac{2(r-1)}{r})s+1, r}} \Vert e^{\phi ^t A^{1/2}} g\Vert _{{\hat{W}}^{1-\gamma ,\frac{2q}{q-1} }}, \end{aligned}$$

for any $1\le q<\frac{d}{\gamma -1}$, where we use Hölder’s inequality on the second factor to obtain the ultimate line.

Next, we make an elementary Bony decomposition by splitting the space of $(\xi ,\eta )$ into the regions $|\xi -\eta | \le \frac{|\eta |}{8}$, $|\eta |\le \frac{|\xi -\eta |}{8}$, and $\frac{1}{8}< \frac{|\xi -\eta |}{|\eta |}<8$.

• If $|\xi -\eta | \le \frac{|\eta |}{8}$, then $|\eta | \sim |\xi |$. So by Young’s inequality followed by application of Lemma 2.1, it holds for any $1\le p\le r$ that

4.19 $$\begin{aligned}&\left( \int _{{\mathbb {R}}^d}\langle \xi \rangle ^{r( (\kappa -\frac{2(r-1)}{r}) s+1)}\left( \int _{\begin{array}{c} |\xi -\eta |\le \frac{|\eta |}{8} \\ |\eta |>1 \end{array}}e^{\phi ^t(1+|\xi -\eta |^s)} |{\hat{f}}(\xi -\eta )| |\eta |^{1-\gamma }e^{\phi ^t(1+|\eta |^s)}|{\hat{g}}(\eta )| d\eta \right) ^r d\xi \right) ^{1/r} \nonumber \\&\quad \lesssim _{r,\kappa ,s,\gamma } \Vert e^{\phi ^t A^{1/2}} f\Vert _{{\hat{L}}^{\frac{pr}{p(r+1)-r}}} \Vert \langle \nabla \rangle ^{(\kappa -\frac{2(r-1)}{r})s+2-\gamma }e^{\phi ^t A^{1/2}}g\Vert _{{\hat{L}}^p} \nonumber \\&\quad \lesssim _{d,r,s,\kappa ,\gamma ,p} \Vert e^{\phi ^t A^{1/2}} f\Vert _{{\hat{W}}^{0,1}} \Vert e^{\phi ^t A^{1/2}} g\Vert _{{\hat{W}}^{\kappa s+2-\gamma ,1}} \textbf{1}_{r=1} \nonumber \\&\qquad + \Vert e^{\phi ^t A^{1/2}} f\Vert _{{\hat{W}}^{0,r}} \Vert e^{\phi ^t A^{1/2}} g\Vert _{{\hat{W}}^{((\kappa -\frac{2(r-1)}{r})s+2+\frac{d(r-1)}{r}-\gamma )+, r}}\textbf{1}_{\begin{array}{c} r>1 \\ p=1 \end{array}} \nonumber \\&\qquad + \Vert e^{\phi ^t A^{1/2}} f\Vert _{{\hat{W}}^{\frac{d(r-1)}{r}+, r}} \Vert e^{\phi ^t A^{1/2}} g\Vert _{{\hat{W}}^{(\kappa - \frac{2(r-1)}{r})s+2-\gamma , r}}\textbf{1}_{\begin{array}{c} r>1 \\ p=r \end{array}} \nonumber \\&\qquad + \Vert e^{\phi ^t A^{1/2}} f\Vert _{{\hat{W}}^{\frac{d(p-1)}{p}+,r}}\Vert e^{\phi ^t A^{1/2}} g\Vert _{{\hat{W}}^{((\kappa -\frac{2(r-1)}{r})s+2-\gamma +\frac{d(r-p)}{rp})+, r}} \textbf{1}_{\begin{array}{c} r>1 \\ 1<p<r \end{array}}. \end{aligned}$$

• If $|\eta |\le \frac{|\xi -\eta |}{8}$, then $|\xi -\eta |\sim |\xi |$. Again using Young’s inequality and Lemma 2.1, it holds for any $1\le \tilde{p}\le r$ that

4.20 $$\begin{aligned}&\left( \int _{{\mathbb {R}}^d}\langle \xi \rangle ^{r( (\kappa -\frac{2(r-1)}{r}) s+1)}\left( \int _{\begin{array}{c} |\eta |\le \frac{|\xi -\eta |}{8} \\ |\eta |>1 \end{array}}e^{\phi ^t(1+|\xi -\eta |^s)} |{\hat{f}}(\xi -\eta )| |\eta |^{1-\gamma }e^{\phi ^t(1+|\eta |^s)}|{\hat{g}}(\eta )| d\eta \right) ^r d\xi \right) ^{1/r} \nonumber \\&\quad \lesssim _{r,\kappa ,s,\gamma } \Vert \langle \nabla \rangle ^{(\kappa -\frac{2(r-1)}{r})s +1} e^{\phi ^t A^{1/2}}f\Vert _{{\hat{L}}^{\frac{\tilde{p}r}{\tilde{p}(r+1)-r}}} \Vert \langle \nabla \rangle ^{1-\gamma }e^{\phi ^t A^{1/2}} g\Vert _{{\hat{L}}^{\tilde{p}}} \nonumber \\&\quad \lesssim _{d,s,\kappa ,\gamma ,r,\tilde{p}} \Vert e^{\phi ^t A^{1/2}} f\Vert _{{\hat{W}}^{\kappa s +1,1}}\Vert e^{\phi ^t A^{1/2}} g\Vert _{{\hat{W}}^{1-\gamma ,1}} \textbf{1}_{r=1} \nonumber \\&\qquad + \Vert e^{\phi ^t A^{1/2}} f\Vert _{{\hat{W}}^{(\kappa -\frac{2(r-1)}{r})s + 1,r}}\Vert e^{\phi ^t A^{1/2}} g\Vert _{{\hat{W}}^{(1-\gamma +\frac{d(r-1)}{r})+,r}}\textbf{1}_{\begin{array}{c} r>1 \\ \tilde{p}=1 \end{array}} \nonumber \\&\qquad + \Vert e^{\phi ^t A^{1/2}} f\Vert _{{\hat{W}}^{((\kappa -\frac{2(r-1)}{r})s + 1+\frac{d(r-1)}{r})+,r}}\Vert e^{\phi ^t A^{1/2}} g\Vert _{{\hat{W}}^{1-\gamma ,r}}\textbf{1}_{\begin{array}{c} r>1 \\ \tilde{p}=r \end{array}} \nonumber \\&\qquad + \Vert e^{\phi ^t A^{1/2}} f\Vert _{{\hat{W}}^{((\kappa -\frac{2(r-1)}{r})s + 1+\frac{d(\tilde{p}-1)}{\tilde{p}})+,r}}\Vert e^{\phi ^t A^{1/2}} g\Vert _{{\hat{W}}^{(1-\gamma + \frac{d(r-\tilde{p})}{r\tilde{p}})+,r}}\textbf{1}_{\begin{array}{c} r>1 \\ 1<\tilde{p}<r \end{array}}. \end{aligned}$$

- If $\frac{1}{8}< \frac{|\xi -\eta |}{|\eta |}<8$, then $\min \{|\xi -\eta |,|\eta |\} \gtrsim |\xi |$. So we can evenly distribute the derivatives between *f* and *g* and use Young’s inequality together with Lemma 2.1 to obtain
  4.21 $$\begin{aligned}&\left( \int _{{\mathbb {R}}^d}\langle \xi \rangle ^{r( (\kappa -\frac{2(r-1)}{r}) s+1)}\left( \int _{\begin{array}{c} \frac{1}{8} \le \frac{|\xi -\eta |}{|\eta |} \le 8 \\ |\eta |>1 \end{array}}e^{\phi ^t(1+|\xi -\eta |^s)} |{\hat{f}}(\xi -\eta )| |\eta |^{1-\gamma }e^{\phi ^t(1+|\eta |^s)}|{\hat{g}}(\eta )| d\eta \right) ^r d\xi \right) ^{1/r} \nonumber \\&\lesssim _{r,\kappa ,s,\gamma } \Vert \langle \nabla \rangle ^{\frac{(\kappa -\frac{2(r-1)}{r})s +2-\gamma }{2}} e^{\phi ^t A^{1/2}}f\Vert _{{\hat{L}}^{\frac{2r}{r+1}}} \Vert \langle \nabla \rangle ^{\frac{(\kappa -\frac{2(r-1)}{r})s +2-\gamma }{2}} e^{\phi ^t A^{1/2}}g\Vert _{{\hat{L}}^{\frac{2r}{r+1}}} \nonumber \\&\lesssim _{d,r,\kappa ,s,\gamma } \Vert e^{\phi ^t A^{1/2}} f\Vert _{{\hat{W}}^{\frac{\kappa s +2-\gamma }{2},1}} \Vert e^{\phi ^t A^{1/2}} g\Vert _{{\hat{W}}^{\frac{\kappa s +2-\gamma }{2},1}} \textbf{1}_{r=1} \nonumber \\&+\Vert e^{\phi ^t A^{1/2}} f\Vert _{{\hat{W}}^{(\frac{(\kappa -\frac{2(r-1)}{r})s +2 + \frac{d(r-1)}{r}-\gamma }{2})+,r}}\Vert e^{\phi ^t A^{1/2}} g\Vert _{{\hat{W}}^{(\frac{(\kappa -\frac{2(r-1)}{r})s +2 + \frac{d(r-1)}{r}-\gamma }{2})+,r}} \textbf{1}_{r>1}. \end{aligned}$$

Combining the estimates (4.18), (4.19), (4.20), (4.21), we see that

4.22 $$\begin{aligned}&\Bigg (\int _{{\mathbb {R}}^d} \langle \xi \rangle ^{(r\kappa - 2(r-1))s+r} \left| \int _{{\mathbb {R}}^d}|\eta |^{1-\gamma } \left| \left( e^{\phi ^t(1+|\xi -\eta |^s)}{\hat{f}}(\xi -\eta )\right) \left( e^{\phi ^t(1+|\eta |^s)}{\hat{g}}(\eta )\right) \right| d\eta \right| ^r d\xi \Bigg )^{1/r} \nonumber \\&\quad \lesssim _{d,s,\gamma ,\kappa ,r,q,p,\tilde{p}} \Vert e^{\phi ^t A^{1/2}}f\Vert _{{\hat{W}}^{(\kappa -\frac{2(r-1)}{r})s+1, r}} \Vert e^{\phi ^t A^{1/2}} g\Vert _{{\hat{W}}^{1-\gamma ,\frac{2q}{q-1}}}\textbf{1}_{\gamma>1} \nonumber \\&\quad + \Bigg (\Vert e^{\phi ^t A^{1/2}} f\Vert _{{\hat{W}}^{0,1}} \Vert e^{\phi ^t A^{1/2}} g\Vert _{{\hat{W}}^{\kappa s+2-\gamma ,1}} + \Vert e^{\phi ^t A^{1/2}} f\Vert _{{\hat{W}}^{\kappa s +1,1}}\Vert e^{\phi ^t A^{1/2}} g\Vert _{{\hat{W}}^{1-\gamma ,1}} \nonumber \\&\quad + \Vert e^{\phi ^t A^{1/2}} f\Vert _{{\hat{W}}^{\frac{\kappa s +2-\gamma }{2},1}} \Vert e^{\phi ^t A^{1/2}} g\Vert _{{\hat{W}}^{\frac{\kappa s +2-\gamma }{2},1}} \Bigg )\textbf{1}_{r=1}\nonumber \\&\quad +\Vert e^{\phi ^t A^{1/2}} f\Vert _{{\hat{W}}^{\frac{d(p-1)}{p}+,r}}\Vert e^{\phi ^t A^{1/2}} g\Vert _{{\hat{W}}^{((\kappa -\frac{2(r-1)}{r})s+2-\gamma +\frac{d(r-p)}{rp})+, r}} \textbf{1}_{\begin{array}{c} r>1 \\ 1<p<r \end{array}}\nonumber \\&\quad + \Vert e^{\phi ^t A^{1/2}} f\Vert _{{\hat{W}}^{((\kappa -\frac{2(r-1)}{r})s + 1+\frac{d(\tilde{p}-1)}{\tilde{p}})+,r}}\Vert e^{\phi ^t A^{1/2}} g\Vert _{{\hat{W}}^{(1-\gamma + \frac{d(r-\tilde{p})}{r\tilde{p}})+,r}}\textbf{1}_{\begin{array}{c} r>1 \\ 1<\tilde{p}<r \end{array}} \nonumber \\&\quad +\Vert e^{\phi ^t A^{1/2}} f\Vert _{{\hat{W}}^{(\frac{(\kappa -\frac{2(r-1)}{r})s +2 + \frac{d(r-1)}{r}-\gamma }{2})+,r}}\Vert e^{\phi ^t A^{1/2}} g\Vert _{{\hat{W}}^{(\frac{(\kappa -\frac{2(r-1)}{r})s +2 + \frac{d(r-1)}{r}-\gamma }{2})+,r}} \textbf{1}_{r>1}. \end{aligned}$$

Above, we have limited ourselves to the cases $r=1$ and $r>1,\ 1<p,\tilde{p} <\infty$ so as to simplify the exposition (the cost is an insignificant $\varepsilon$ loss at the endpoint exponents). In order to obtain the desired estimate (4.16), we need the maximum Sobolev index of the norms in (4.22) to be $<(\kappa +\frac{2}{r})s$. This leads us to make the following assumptions on the parameters $d,s,\kappa ,\gamma ,r,p,\tilde{p}$.

1. $$r=1$$
  - $$\kappa s +2-\gamma \le (\kappa +2)s$$,
  - $$\max \{\kappa s+1,1-\gamma \} \le (\kappa +2)s$$,
  - $$\frac{\kappa s+2-\gamma }{2} \le (\kappa +2)s$$.
2. $$r>1$$
  - $$(\kappa - \frac{2(r-1)}{r})s + 1 \le (\kappa + \frac{2}{r})s$$;
  - there exists $p\in (1,r)$ such that $\max \{\frac{d(p-1)}{p}, (\kappa - \frac{2(r-1)}{r})s + 2-\gamma + \frac{d(r-p)}{rp}\} < (\kappa + \frac{2}{r})s$;
  - there exists $\tilde{p}\in (1,r)$ such that $\max \{(\kappa -\frac{2(r-1)}{r})s + 1+\frac{d(\tilde{p}-1)}{\tilde{p}},1-\gamma + \frac{d(r-\tilde{p})}{r\tilde{p}} \} < (\kappa +\frac{2}{r})s$;
  - $$\frac{(\kappa -\frac{2(r-1)}{r})s +2 + \frac{d(r-1)}{r}-\gamma }{2} < (\kappa +\frac{2}{r})s$$.

Let us analyze the above conditions.

For (U1)a, observe

4.23 $$\begin{aligned} \kappa s + 2-\gamma \le (\kappa +2)s \Longleftrightarrow \frac{2-\gamma }{2}\le s. \end{aligned}$$

Since $\gamma >0$ by assumption, we can always choose *s* sufficiently close to 1, so that this condition holds. For (U1)b, the inequality is equivalent to

4.24 $$\begin{aligned} \frac{1}{2}\le s \quad \text {and} \quad 1-\gamma -2s \le \kappa s. \end{aligned}$$

The first inequality is true by assumption, and the second inequality holds by taking $\kappa$ sufficiently large depending on given $\gamma ,s$. (U2)c is equivalent to

4.25 $$\begin{aligned} 2-\gamma -4s \le \kappa s, \end{aligned}$$

which holds by taking $\kappa$ sufficiently large depending on given $s,\gamma$. (U2)a is equivalent to

4.26 $$\begin{aligned} -\frac{2(r-1)s}{r} + 1 \le \frac{2s}{r} \quad \Longleftrightarrow \quad \frac{1}{2} \le s, \end{aligned}$$

which holds by assumption. For (U2)b, observe that

4.27 $$\begin{aligned} \frac{d(p-1)}{p}< (\kappa + \frac{2}{r})s \quad \Longleftrightarrow \quad d-(\kappa + \frac{2}{r})s < \frac{d}{p} \end{aligned}$$

and

4.28 $$\begin{aligned} (\kappa - \frac{2(r-1)}{r})s + 2-\gamma + \frac{d(r-p)}{rp}< (\kappa + \frac{2}{r})s \quad \Longleftrightarrow \quad \frac{d}{p} < \frac{d}{r} +\gamma +2s-2. \end{aligned}$$

Thus, it is possible to find such a $p\in (1,r)$ if and only if

4.29 $$\begin{aligned} d-(\kappa + \frac{2}{r})s< \frac{d}{r} +\gamma +2s-2\quad \Longleftrightarrow \quad d-\frac{2s}{r}-\frac{d}{r}-\gamma -2s+2 < \kappa s, \end{aligned}$$

which holds by taking $\kappa$ sufficiently large depending on given $d,\gamma ,r,s$. For (U2)c, observe

4.30 $$\begin{aligned} \left( \kappa -\frac{2(r-1)}{r}\right) s + 1+\frac{d(\tilde{p}-1)}{\tilde{p}}<\left( \kappa +\frac{2}{r}\right) s \quad \Longleftrightarrow \quad d+1-2s < \frac{d}{\tilde{p}} \end{aligned}$$

and

4.31 $$\begin{aligned} 1-\gamma +\frac{d(r-\tilde{p})}{r\tilde{p}}< \left( \kappa +\frac{2}{r}\right) s \quad \Longleftrightarrow \quad \frac{d}{\tilde{p}} < \kappa s + \frac{2s+d}{r}+\gamma -1.\nonumber \\ \end{aligned}$$

It is possible to find such a $\tilde{p}\in (1,r)$ if and only if

4.32 $$\begin{aligned} d+1-2s< \kappa s + \frac{2s+d}{r}+\gamma -1 \quad \Longleftrightarrow \quad d+2-2s-\frac{2s+d}{r} - \gamma < \kappa s,\nonumber \\ \end{aligned}$$

which holds by taking $\kappa$ sufficiently large depending on given $d,\gamma ,r,s$. Lastly, (U2)d is equivalent to

4.33 $$\begin{aligned} d+2 -\gamma -2s-\frac{d+2s}{r}<\kappa s, \end{aligned}$$

which holds by taking $\kappa$ sufficiently large depending on given $d,\gamma ,r,s$.

Next, we observe that in order to obtain the desired estimate (4.16), we need the minimum Sobolev index of the norms appearing in (4.22) to be $\le \kappa s$. This leads us to make the following additional assumptions on the parameters $d,s,\kappa ,\gamma ,r,p,\tilde{p}$:

1. $$r=1$$
  - $$1-\gamma \le \kappa s$$
  - $$\frac{\kappa s+2-\gamma }{2} \le \kappa s$$
2. $$r>1$$
  - there exists $p\in (1,r)$ such that $\min \{\frac{d(p-1)}{p}, (\kappa - \frac{2(r-1)}{r})s + 2-\gamma + \frac{d(r-p)}{rp}\} < \kappa s$
  - there exists $\tilde{p}\in (1,r)$ such that $\min \{(\kappa - \frac{2(r-1)}{r})s + 1+\frac{d(\tilde{p}-1)}{\tilde{p}}, 1-\gamma + \frac{d(r-\tilde{p})}{r\tilde{p}}\} < \kappa s$
  - $$\frac{(\kappa - \frac{2(r-1)}{r})s+2+\frac{d(r-1)}{r}-\gamma }{2} < \kappa s$$.

Let us analyze the preceding assumptions.

For any $\frac{1}{2}<s\le 1$, (L1)a always holds if we assume $2(1-\gamma ) \le \kappa$. For (L1)b,

4.34 $$\begin{aligned} \frac{\kappa s+2-\gamma }{2} \le \kappa s \quad \Longleftrightarrow \quad 2-\gamma \le \kappa s, \end{aligned}$$

which is ensured by taking $\kappa$ sufficiently large depending on given $\gamma ,s$. For (L2)a, we need

4.35 $$\begin{aligned} \frac{d(p-1)}{p}< \kappa s \quad \text {or} \quad (\kappa - \frac{2(r-1)}{r})s + 2-\gamma + \frac{d(r-p)}{rp} < \kappa s. \end{aligned}$$

Since $p<r$ and therefore $\frac{d(p-1)}{p} < \frac{d(r-1)}{r}$, the first inequality is valid if $\frac{d(r-1)}{r}<\kappa s$, which holds by taking $\kappa$ sufficiently large depending on given *d*, *r*, *s*. For the second inequality, we see

4.36 $$\begin{aligned} \left( \kappa - \frac{2(r-1)}{r}\right) s + 2-\gamma + \frac{d(r-p)}{rp}< \kappa s \quad \Longleftrightarrow \quad 2-2s-\gamma + \frac{d}{p}+\frac{(2s-d)}{r} < 0. \end{aligned}$$

The left-hand side of the second inequality is maximized by choosing $p=1$, so it would suffice to assume $d,\gamma ,s,r$ satisfy

4.37 $$\begin{aligned} 2-2s-\gamma +d+\frac{(2s-d)}{r}<0. \end{aligned}$$

For (L2)b, we need

4.38 $$\begin{aligned} \left( \kappa - \frac{2(r-1)}{r}\right) s + 1+\frac{d(\tilde{p}-1)}{\tilde{p}}< \kappa s \quad \text {or} \quad 1-\gamma + \frac{d(r-\tilde{p})}{r\tilde{p}} < \kappa . \end{aligned}$$

The first inequality is equivalent to

4.39 $$\begin{aligned} d+1-2s +\frac{2s}{r} - \frac{d}{\tilde{p}} < 0. \end{aligned}$$

The left-hand side is maximized by choosing $\tilde{p}=r$, therefore it suffices to assume

4.40 $$\begin{aligned} d+1-2s+\frac{(2s-d)}{r}<0. \end{aligned}$$

The second inequality is equivalent to

4.41 $$\begin{aligned} 1-\gamma +\frac{d}{\tilde{p}}-\frac{d}{r}<\kappa s, \end{aligned}$$

the left-hand side of which is maximized by choosing $\tilde{p}=1$. So, it would suffice to assume

4.42 $$\begin{aligned} 1-\gamma + d-\frac{d}{r}<\kappa s, \end{aligned}$$

which is seen to hold by choosing $\kappa$ sufficiently large depending on given $d,\gamma ,r,s$. For (L2)c, we observe that the inequality is equivalent to

4.43 $$\begin{aligned} d+2-\gamma -2s+\frac{2s-d}{r} < \kappa s, \end{aligned}$$

which is valid provided that $\kappa$ is sufficiently large depending on given $d,\gamma ,r,s$.

The preceding sets of assumptions tell us that given $d,\gamma ,r,s$, there is a threshold $\kappa _0$ depending on $d,\gamma ,r,s$, such that for all $\kappa >\kappa _0$, the right-hand side of (4.22) is controlled by

4.44 $$\begin{aligned}&\Vert e^{\phi ^t A^{1/2}}f\Vert _{{\hat{W}}^{(\kappa -\frac{2(r-1)}{r})s+1, r}} \Vert e^{\phi ^t A^{1/2}} g\Vert _{{\hat{W}}^{1-\gamma , \frac{2q}{q-1}}}\textbf{1}_{\gamma >1} \nonumber \\&\quad + \Vert e^{\phi ^t A^{1/2}}f\Vert _{{\hat{W}}^{(\kappa + \frac{2}{r})s,r}} \Vert e^{\phi ^t A^{1/2}}g\Vert _{{\hat{W}}^{\kappa s,r}} + \Vert e^{\phi ^t A^{1/2}}f\Vert _{{\hat{W}}^{\kappa s,r}}\Vert e^{\phi ^t A^{1/2}}g\Vert _{{\hat{W}}^{(\kappa + \frac{2}{r})s,r}}. \end{aligned}$$

Recalling the starting inequality (4.17), we see that the proof is complete. $\square$

We are now prepared to prove Proposition 4.1.

#### Proof of Proposition 4.1

Given $1< q < \frac{d}{\gamma -1}$, set $q':=\frac{q}{q-1}$. We first show that the quantity

4.45 $$\begin{aligned} \Vert e^{\phi ^t A^{1/2}}\mu ^t\Vert _{{\hat{W}}^{\kappa s, 2q'}}^{2q'} \end{aligned}$$

is strictly decreasing on an interval [0, *T*], provided that $\kappa$ sufficiently large depending on $q'$ and the higher norm $\Vert e^{\phi ^t A^{1/2}}\mu ^t\Vert _{{\hat{W}}^{(\kappa + \frac{1}{q'})s, 2q'}}$ remains finite on [0, *T*]. Indeed, let $\kappa _{0,2q'}$ denote the threshold given by Lemma 4.4 with $r=2q'$, and suppose that $\kappa >\kappa _{0,2q'}$. Using Hölder’s inequality and Lemmas 4.3 and 4.4, we see that

4.46 $$\begin{aligned}&\int _{{\mathbb {R}}^d} \left| e^{\phi ^t (1+|\xi |^s)}\langle \xi \rangle ^{\kappa s} {\hat{\mu }}^t(\xi ) \right| ^{2q'-1}\langle \xi \rangle ^{\kappa s} e^{\phi ^t(1+|\xi |^s)}|{\mathcal {F}}(B^t(\mu ^t,\mu ^t))(\xi )|d\xi \nonumber \\&\quad \le C|{\mathbb {M}}|\Vert e^{\phi ^t A^{1/2}} \mu ^t\Vert _{{\hat{W}}^{(\kappa +\frac{1}{q'})s,2q'}}^{2q'-1}\Bigg (\Vert e^{\phi ^t A^{1/2}}\mu ^t\Vert _{{\hat{W}}^{(\kappa -2+\frac{1}{q'})s+1, 2q'}} \Vert e^{\phi ^t A^{1/2}} \mu ^t\Vert _{{\hat{W}}^{1-\gamma ,2q'}}\textbf{1}_{\gamma >1} \nonumber \\&\quad + \Vert e^{\phi ^t A^{1/2}}\mu ^t\Vert _{{\hat{W}}^{(\kappa + \frac{1}{q'})s,2q'}} \Vert e^{\phi ^t A^{1/2}}\mu ^t\Vert _{{\hat{W}}^{\kappa s,2q'}}\Bigg ) \end{aligned}$$

where the constant $C>0$ depends only on $d,\gamma ,q,s,\kappa$. Applying this bound to the differential identity (4.11) (with *r* replaced by $2q'$), it follows that

4.47 $$\begin{aligned}&\frac{d}{dt} \frac{1}{2q'} \Vert e^{\phi ^t A^{1/2}} \mu ^t\Vert _{{\hat{W}}^{\kappa s,2q'}}^{2q'} \le -\left( \frac{\nu ^2}{2} -\beta \right) \Vert e^{\phi ^t A^{1/2}}\mu ^t\Vert _{{\hat{W}}^{(\kappa +\frac{1}{q'})s,2q'}}^{2q'} \nonumber \\&\quad +C|{\mathbb {M}}|\Vert e^{\phi ^t A^{1/2}} \mu ^t\Vert _{{\hat{W}}^{(\kappa +\frac{1}{q'})s,2q'}}^{2q'-1}\Bigg (\Vert e^{\phi ^t A^{1/2}}\mu ^t\Vert _{{\hat{W}}^{(\kappa -2+\frac{1}{q'})s+1, 2q'}} \Vert e^{\phi ^t A^{1/2}} g\Vert _{{\hat{W}}^{1-\gamma ,2q'}}\textbf{1}_{\gamma >1} \nonumber \\&\quad + \Vert e^{\phi ^t A^{1/2}}\mu ^t\Vert _{{\hat{W}}^{(\kappa + \frac{1}{q'})s,2q'}} \Vert e^{\phi ^t A^{1/2}}\mu ^t\Vert _{{\hat{W}}^{\kappa s,2q'}} \Bigg ). \end{aligned}$$

Note that since $s>\frac{1}{2}$ by assumption, $(\kappa -2+\frac{1}{q'})s + 1 < (\kappa + \frac{1}{q'})s$. Thus, the right-hand side of the preceding inequality is $\le$

4.48 $$\begin{aligned} \Vert e^{\phi ^t A^{1/2}}\mu ^t\Vert _{{\hat{W}}^{(\kappa +\frac{1}{q'})s,2q'}}^{2q'}\Bigg (C|{\mathbb {M}}|\Vert e^{\phi ^t A^{1/2}}\mu ^t\Vert _{{\hat{W}}^{\kappa s,2q'}} -\left( \frac{\nu ^2}{2} -\beta \right) \Bigg ). \end{aligned}$$

We now want to show that if $\Vert e^{\alpha A^{1/2}}\mu ^0\Vert _{{\hat{W}}^{\kappa s, 2q'}} < \frac{\nu ^2 - 2\beta }{2C|{\mathbb {M}}|}$ and the first factor remains finite on [0, *T*], then $\Vert e^{\phi ^t A^{1/2}}\mu ^t\Vert _{{\hat{W}}^{\kappa s, 2q'}}$ is strictly decreasing on [0, *T*]. To do this, we use a continuity argument.

Let $T_*$ denote the maximal time in [0, *T*] such that

4.49 $$\begin{aligned} \Vert e^{\phi ^t A^{1/2}}\mu ^t\Vert _{{\hat{W}}^{\kappa s, 2q'}} < \frac{\nu ^2-2\beta }{2C|{\mathbb {M}}|} \qquad \forall t\in [0,T_*). \end{aligned}$$

Such a $$T_*$$ exists and is positive since the preceding inequality is true at $$t=0$$ by assumption and the function $$t\mapsto \Vert e^{\phi ^t A^{1/2}}\mu ^t\Vert _{{\hat{W}}^{\kappa s,2q'}}$$ is continuous on [0, *T*]. (4.49) together with (4.48) imply that the right-hand side of (4.47) is negative on $$[0,T_*)$$. Hence, the function $$t\mapsto \Vert e^{\phi ^t A^{1/2}}\mu ^t\Vert _{{\hat{W}}^{\kappa s, 2q'}}$$ is strictly decreasing on $$[0,T_*)$$, implying

4.50 $$\begin{aligned} \Vert e^{\phi ^t A^{1/2}}\mu ^{T_*}\Vert _{{\hat{W}}^{\kappa s, 2q'}}<\Vert e^{\phi ^t A^{1/2}}\mu ^0\Vert _{{\hat{W}}^{\kappa s, 2q'}} < \frac{\nu ^2-2\beta }{2C|{\mathbb {M}}|}. \end{aligned}$$

This inequality implies by maximality that $T_*=T$, and therefore strict monotonicity holds on [0, *T*].

We next show that this monotonicity property of the norm $\Vert e^{\phi ^t A^{1/2}}\mu ^t\Vert _{{\hat{W}}^{\kappa s, 2q'}}$ also implies a monotonicity property for the norm $\Vert e^{\phi ^t A^{1/2}}\mu ^t\Vert _{{\hat{W}}^{\tilde{\kappa } s,r}}$, for appropriate $\tilde{\kappa }$, provided that $\Vert e^{\alpha A^{1/2}}\mu ^0\Vert _{{\hat{W}}^{\tilde{\kappa } s, r}}$ is sufficiently small. If $\gamma \le 1$, then this step is unnecessary and the argument given above suffices with $2q'$ replaced by *r*.

Let $\kappa _{0,r}$ denote the regularity threshold given by Lemma 4.4, and let $\kappa _r > \kappa _{0,r}$. Again using Hölder’s inequality and Lemmas 4.3 and 4.4, we see that

4.51 $$\begin{aligned}&\int _{{\mathbb {R}}^d} \left| e^{\phi ^t (1+|\xi |^s)}\langle \xi \rangle ^{\kappa _r s} {\hat{\mu }}^t(\xi ) \right| ^{r-1}\langle \xi \rangle ^{\kappa _r s} e^{\phi ^t(1+|\xi |^s)}|{\mathcal {F}}(B^t(\mu ^t,\mu ^t))(\xi )|d\xi \nonumber \\&\quad \le C_{r,q}|{\mathbb {M}}|\Vert e^{\phi ^t A^{1/2}} \mu ^t\Vert _{{\hat{W}}^{(\kappa _r+\frac{2}{r})s,r}}^{r-1}\Bigg (\Vert e^{\phi ^t A^{1/2}}\mu ^t\Vert _{{\hat{W}}^{(\kappa _r-2+\frac{2}{r})s+1, r}} \Vert e^{\phi ^t A^{1/2}} \mu ^t\Vert _{{\hat{W}}^{1-\gamma ,2q'}}\textbf{1}_{\gamma >1} \nonumber \\&\quad + \Vert e^{\phi ^t A^{1/2}}\mu ^t\Vert _{{\hat{W}}^{(\kappa _r + \frac{2}{r})s,r}} \Vert e^{\phi ^t A^{1/2}}\mu ^t\Vert _{{\hat{W}}^{\kappa _r s,r}} \Bigg ), \end{aligned}$$

where the constant $C_{r,q}>0$ depends only on $d,\gamma ,r,q,s,\kappa _r$. We use the subscript *r* to emphasize the dependence on *r*, as we shall momentarily invoke another constant and regularity parameter depending on *q*. Applying the preceding bound to the differential identity (4.11) it follows that

4.52 $$\begin{aligned}&\frac{d}{dt}\frac{1}{r}\Vert e^{\phi ^t A^{1/2}}\mu ^t\Vert _{{\hat{W}}^{\kappa _r s,r}}^r \le \Bigg (C_{r,q}|{\mathbb {M}}| \Big (\Vert e^{\phi ^t A^{1/2}}\mu ^t\Vert _{{\hat{W}}^{\kappa _r s,r}} + \Vert e^{\phi ^t A^{1/2}} \mu ^t\Vert _{{\hat{W}}^{1-\gamma ,2q'}}\textbf{1}_{\gamma >1}\Big )\nonumber \\&\quad -\left( \frac{\nu ^2}{2} -\beta \right) \Bigg ) \Vert e^{\phi ^t A^{1/2}}\mu ^t\Vert _{{\hat{W}}^{(\kappa _r+\frac{2}{r})s,r}}^r. \end{aligned}$$

Since $1-\gamma < 0$ if $\gamma >1$, we know that there is a constant $C_q$ depending on $d,\gamma ,q,s,\kappa _q$, for $\kappa _q >\kappa _{0,2q'}$, such that if

4.53 $$\begin{aligned} \Vert e^{\alpha A^{1/2}}\mu ^0\Vert _{{\hat{W}}^{\kappa _q s, 2q'}}< \frac{\nu ^2-2\beta }{2C_q|{\mathbb {M}}|} \quad \text {and} \quad \Vert \mu \Vert _{C_T^0{\textsf{G}}_{\phi }^{(\kappa _q+\frac{1}{q'}), 2q'}} < \infty , \end{aligned}$$

then $\Vert e^{\phi ^t A^{1/2}}\mu ^t\Vert _{{\hat{W}}^{\kappa _q s, 2q'}}$ is strictly decreasing on [0, *T*]. A fortiori,

4.54 $$\begin{aligned} \Vert e^{\phi ^t A^{1/2}}\mu ^t\Vert _{{\hat{W}}^{1-\gamma ,2q'}} \le \Vert e^{\alpha A^{1/2}}\mu ^0\Vert _{{\hat{W}}^{\kappa _q, 2q'}} \qquad \forall t\in [0,T]. \end{aligned}$$

Therefore, suppose that

4.55 $$\begin{aligned}&\Vert e^{\alpha A^{1/2}} \mu ^0 \Vert _{{\hat{W}}^{\kappa _q s, 2q'}} < \min \left\{ \frac{\nu ^2-2\beta }{2C_q|{\mathbb {M}}|}, \frac{\nu ^2-2\beta }{2C_{r,q}|{\mathbb {M}}|}\right\} , \end{aligned}$$

4.56 $$\begin{aligned}&\quad \Vert e^{\alpha A^{1/2}} \mu ^0 \Vert _{{\hat{W}}^{\kappa _r s, r}} < \frac{\nu ^2-2\beta }{2C_{r,q}|{\mathbb {M}}|} - \Vert e^{\alpha A^{1/2}} \mu ^0 \Vert _{{\hat{W}}^{\kappa _q s, 2q'}}. \end{aligned}$$

Under these assumptions, it follows by repeating the continuity argument from above that the quantity $\Vert e^{\phi ^t A^{1/2}}\mu ^t\Vert _{{\hat{W}}^{\kappa _r s, r}}$ is strictly decreasing on [0, *T*]. With this last bit, the proof of Proposition 4.1 is complete. $\square$

### Proof of Theorem 1.2

We now use the local well-posedness established by Proposition 3.1 together with the monotonicity of the Gevrey norm established by Proposition 4.1 in order to show that with high probability, solutions in the class we consider are global. Moreover, their Gevrey norm strictly decreases as time $t\rightarrow \infty$. This then proves Theorem 1.2. To show the desired result, we use a refined reformulation of the iterative argument from [14, Section 5].

Let us first present the case $0<\gamma \le 1$, which is simpler due to not needing a two-tiered norm. Fix $\epsilon >0$ and suppose that $\mu ^0\in {\textsf{G}}_{\alpha +\epsilon }^{\sigma _0,r}$ for $\sigma _0$ above the regularity threshold $\kappa _{0,r}$ given by Proposition 4.1. Throughout this subsection, we assume that the parameters $d,\gamma ,r,s,\sigma _0,\alpha ,\beta ,\nu$ satisfy all the constraints of Theorem 1.2. We also assume that

4.57 $$\begin{aligned} 0< \Vert \mu ^0\Vert _{{\textsf{G}}_{\alpha +\epsilon }^{\sigma _0,r}} < \frac{\nu ^2-2\beta }{C_{mon}|{\mathbb {M}}|}, \end{aligned}$$

where $C_{mon}=C_r>0$ is the constant from Proposition 4.1. The strict positivity assumption is because $\mu ^0=0$ implies $\mu$ is identically zero, and therefore strict monotonicity does not hold in this trivial case. Assuming a realization of *W* from $\Omega _{\alpha ,\beta ,\nu }$ and given $r\ge 1$ sufficiently small depending on $d,\gamma ,s$, Proposition 3.1 implies that for any $0<\sigma <\frac{2s-1}{s}$, with $1-\gamma \le \sigma s$, sufficiently large depending on $d,\gamma ,s,r$, there is a maximal solution $\mu$ to the Cauchy problem (3.1) with lifespan $[0,T_{\max ,\sigma ,\epsilon })$, such that $\mu$ belongs to $C_T^0{\textsf{G}}_{\phi +\epsilon }^{\sigma ,r}$ for any $0\le T<T_{\max ,\sigma ,\epsilon }$. Our main lemma to conclude global existence is the following result relating the lifespan of $\mu ^t$ in ${\textsf{G}}_{\phi ^t+\epsilon }^{\sigma ,r}$ to the lifespan of $\mu$ in the *larger space* ${\textsf{G}}_{\phi ^t+\epsilon '}^{\sigma ,r}$, for any $\epsilon '\in [0,\epsilon )$.

#### Lemma 4.5

Let $\mu$ be as above. There exists a constant $C>0$ depending on $d,\gamma ,r,s,\sigma ,\beta ,\nu$ such that for any $0\le \epsilon _2<\epsilon _1\le \epsilon$, the maximal times of existence $T_{\max ,\sigma ,\epsilon _1}, T_{\max ,\sigma ,\epsilon _2}$ of $\mu ^t$ as taking values in ${\textsf{G}}_{\phi ^t+\epsilon _1}^{\sigma ,r}, {\textsf{G}}_{\phi ^t+\epsilon _2}^{\sigma ,r}$, respectively, satisfy the inequality

4.58 $$\begin{aligned} T_{\max ,\sigma ,\epsilon _2} \ge T_{\max ,\sigma ,\epsilon _1} + C\left( |{\mathbb {M}}|\Vert \mu ^0\Vert _{{\textsf{G}}_{\alpha +\epsilon }^{\sigma _0,r}}\right) ^{-\frac{2s}{2s-\sigma s-1}}. \end{aligned}$$

#### Proof

Fix $0<\epsilon _2 <\epsilon _1\le \epsilon$. For any $\sigma '\ge \sigma$, it follows from Lemma 3.5 that $\mu$ is also a solution in $C_T^0{\textsf{G}}_{\phi +\epsilon _2}^{\sigma ',r}$ for any $0\le T<T_{\max ,\sigma ,\epsilon _1}$. Furthermore, we have the quantitative bound

4.59 $$\begin{aligned} \Vert \mu ^t\Vert _{{\textsf{G}}_{\phi ^t+\epsilon _2}^{\sigma ',r}} \le \frac{\lceil {\sigma '-\sigma }\rceil !}{(\epsilon _1-\epsilon _2)^{\lceil {\sigma '-\sigma }\rceil }} \Vert \mu ^t\Vert _{{\textsf{G}}_{\phi ^t+\epsilon _1}^{\sigma ,r}} \qquad \forall \ 0\le t<T_{\max ,\sigma ,\epsilon _1}. \end{aligned}$$

Choose $\sigma '$ such that

4.60 $$\begin{aligned} \sigma _0 \ge \sigma ' > \kappa _{0,r}, \end{aligned}$$

where $\kappa _{0,r}$ is the regularity threshold of Proposition 4.1. Using the assumption (4.57), we can then apply Proposition 4.1 to conclude that the function $t\mapsto \Vert \mu ^t\Vert _{{\textsf{G}}_{\phi ^t+\epsilon _2}^{\sigma ',r}}$ is strictly decreasing on the interval [0, *T*] for any $T<T_{\max ,\sigma ,\epsilon _1}$. In particular, since we can ensure that $\sigma \le \sigma '$, it follows from this monotonicity and Lemma 3.5 that

4.61 $$\begin{aligned} \Vert \mu \Vert _{C_T^0{\textsf{G}}_{\phi +\epsilon _2}^{\sigma ,r}} \le \Vert \mu \Vert _{C_T^0{\textsf{G}}_{\phi +\epsilon _2}^{\sigma ',r}} \le \Vert \mu ^0\Vert _{{\textsf{G}}_{\alpha +\epsilon _2}^{\sigma ',r}} \le \Vert \mu ^0\Vert _{{\textsf{G}}_{\alpha +\epsilon }^{\sigma _0,r}} \qquad \forall \ 0\le T<T_{\max ,\sigma ,\epsilon _1}.\nonumber \\ \end{aligned}$$

Let $C_{lwp,\sigma }$ be the constant from Proposition 3.1, and choose $T_* < T_{\max ,\sigma ,\epsilon _1}$ so that

4.62 $$\begin{aligned} T_{\max ,\sigma ,\epsilon _1}-T_* \le \frac{C_{lwp,\sigma }\left( |{\mathbb {M}}| \Vert \mu ^0\Vert _{{\textsf{G}}_{\alpha +\epsilon }^{\sigma _0,r}}\right) ^{-\frac{2s}{2s-\sigma s-1}}}{2}. \end{aligned}$$

Thus by relabeling time, we can apply Proposition 3.1 once more, but with initial datum $\mu ^{T_*}$, to find that $\mu$ belongs to $C_{T_0}^0{\textsf{G}}_{\phi +\epsilon _2}^{\sigma ,r}$, where

4.63 $$\begin{aligned} T_0&= T_* + C_{lwp,\sigma }(|{\mathbb {M}}| \Vert \mu ^0\Vert _{{\textsf{G}}_{\alpha +\epsilon _2}^{\sigma ,r}})^{-\frac{2s}{2s-\sigma s-1}} \nonumber \\&= (T_*-T_{\max ,\sigma ,\epsilon _1}) + T_{\max ,\sigma ,\epsilon _1} + C_{lwp,\sigma }(|{\mathbb {M}}| \Vert \mu ^0 \Vert _{{\textsf{G}}_{\alpha +\epsilon _2}^{\sigma ,r}})^{-\frac{2s}{2s-\sigma s-1}} \nonumber \\&\ge T_{\max ,\sigma ,\epsilon _1} + \frac{C_{lwp,\sigma }(|{\mathbb {M}}| \Vert \mu ^0\Vert _{{\textsf{G}}_{\alpha +\epsilon }^{\sigma _0,r}})^{-\frac{2s}{2s-\sigma s-1}}}{2}, \end{aligned}$$

where we have used that $\Vert \mu ^0\Vert _{{\textsf{G}}_{\alpha +\epsilon _2}^{\sigma ,r}} \le \Vert \mu ^0\Vert _{{\textsf{G}}_{\alpha +\epsilon _2}^{\sigma _0,r}}$. Taking $C=\frac{C_{lwp,\sigma }}{2}$, the preceding inequality is exactly what we need to show. $\square$

*Proof of Theorem* 1.2*for* $\gamma \le 1$. Fix $0<\epsilon '<\epsilon$. Let $\sigma ,\sigma _0$ be as above. If $T_{\max , \sigma , \epsilon '}<\infty$, then let $n\in {\mathbb {N}}$ be such that $n C (|{\mathbb {M}}|\Vert \mu ^0\Vert _{{\textsf{G}}_{\alpha +\epsilon }^{\sigma _0,r}})^{-\frac{2s}{2s-\sigma s-1}}$ satisfies the inequality

4.64 $$\begin{aligned} n C(|{\mathbb {M}}|\Vert \mu ^0\Vert _{{\textsf{G}}_{\alpha +\epsilon }^{\sigma _0,r}})^{-\frac{2s}{2s-\sigma s-1}} > T_{\max ,\sigma ,\epsilon '}-T_{\max ,\sigma ,\epsilon }, \end{aligned}$$

where *C* is the same constant as in the inequality (4.58). We observe from Lemma 4.5 that

4.65 $$\begin{aligned} T_{\max ,\sigma ,\epsilon '} - T_{\max ,\sigma ,\epsilon }&= \sum _{j=0}^{n-1} \left( T_{\max , \sigma ,\epsilon - \frac{(j+1) (\epsilon -\epsilon ')}{n}} - T_{\max ,\sigma ,\epsilon -\frac{j(\epsilon -\epsilon ')}{n}}\right) \nonumber \\&\ge \sum _{j=0}^{n-1} C(|{\mathbb {M}}|\Vert \mu ^0\Vert _{{\textsf{G}}_{\alpha +\epsilon }^{\sigma _0,r}})^{-\frac{2s}{2s-\sigma s-1}}\nonumber \\&> T_{\max ,\sigma ,\epsilon '}-T_{\max ,\sigma ,\epsilon }, \end{aligned}$$

which is a contradiction. Thus, $T_{\max ,\sigma ,\epsilon '}=\infty$.

We have shown that for any $0<\epsilon '<\epsilon$ and any $0<\sigma <\frac{2s-1}{s}$ sufficiently large depending on $d,\gamma ,s,r$, it holds that $\Vert \mu \Vert _{C_T^0{\textsf{G}}_{\phi +\epsilon '}^{\sigma ,r}}<\infty$ for all $T>0$. Using the arbitrariness of $\epsilon '$, we see from Lemma 3.5 that for any $T>0$, $\Vert \mu \Vert _{C_T^0{\textsf{G}}_{\phi +\epsilon '}^{\sigma _0+\frac{2}{r},r}} < \infty$. Using that

4.66 $$\begin{aligned} \Vert \mu ^0\Vert _{{\textsf{G}}_{\alpha +\epsilon '}^{\sigma _0,r}} \le \Vert \mu ^0\Vert _{{\textsf{G}}_{\alpha +\epsilon }^{\sigma _0,r}} < \frac{\nu ^2-2\beta }{C_{mon}|{\mathbb {M}}|} \end{aligned}$$

by assumption (4.57), where $C_{mon}>0$ is the constant from Proposition 4.1, we can apply Proposition 4.1 on the interval [0, *T*] to obtain that the function $t\mapsto \Vert \mu ^t\Vert _{{\textsf{G}}_{\phi ^t+\epsilon '}^{\sigma _0,r}}$ is strictly decreasing on [0, *T*]. Since $T>0$ was arbitrary, we see that this monotonicity property holds on the entire interval $[0,\infty )$.

Finally, we show that $\mu$ actually belongs to $C_\infty ^0{\textsf{G}}_{\phi +\epsilon }^{\sigma _0,r}$ and that the decreasing property holds on $[0,\infty )$. Note that there is no longer a loss in the Gevrey index value (i.e. $\epsilon '=\epsilon$). To this end, we observe from the result of the preceding paragraph and $\Vert \cdot \Vert _{{\textsf{G}}_{\alpha +\epsilon '}^{\sigma _0,r}} \le \Vert \cdot \Vert _{{\textsf{G}}_{\alpha +\epsilon }^{\sigma _0,r}}$, for $\epsilon '<\epsilon$, that for any $t\ge 0$,

4.67 $$\begin{aligned} \Vert \mu ^t\Vert _{{\textsf{G}}_{\phi ^t+\epsilon '}^{\sigma _0,r}} \le \Vert \mu ^0\Vert _{{\textsf{G}}_{\alpha +\epsilon '}^{\sigma _0,r}} \le \Vert \mu ^0\Vert _{{\textsf{G}}_{\alpha +\epsilon }^{\sigma _0,r}} < \infty . \end{aligned}$$

Unpacking the definition of the left-hand side and appealing to monotone convergence theorem, it follows that

4.68 $$\begin{aligned} \Vert \mu ^t\Vert _{{\textsf{G}}_{\phi ^t+\epsilon }^{\sigma _0,r}} = \lim _{\epsilon '\rightarrow \epsilon ^{-}} \left( \int _{{\mathbb {R}}^d} \left| \langle \xi \rangle ^{\sigma _0} e^{(\phi ^t+\epsilon ')(1+|\xi |^s)}{\hat{\mu }}^t(\xi ) \right| ^{r} d\xi \right) ^{1/r} \le \Vert \mu ^0\Vert _{{\textsf{G}}_{\alpha +\epsilon }^{\sigma _0,r}}.\nonumber \\ \end{aligned}$$

Similarly, for any $t_2\ge t_1\ge 0$,

4.69 $$\begin{aligned} \Vert \mu ^{t_2}\Vert _{{\textsf{G}}_{\phi ^{t_2}+\epsilon }^{\sigma _0,r}} = \lim _{\epsilon '\rightarrow \epsilon ^-} \Vert \mu ^{t_2} \Vert _{{\textsf{G}}_{\phi ^{t_2}+\epsilon '}^{\sigma _0,r}}\le \lim _{\epsilon '\rightarrow \epsilon ^-} \Vert \mu ^{t_1} \Vert _{{\textsf{G}}_{\phi ^{t_1}+\epsilon '}^{\sigma _0,r}} = \Vert \mu ^{t_1} \Vert _{{\textsf{G}}_{\phi ^{t_1}+\epsilon }^{\sigma _0,r}}, \end{aligned}$$

where the inequality is strict if $t_2>t_1$. This completes the proof of Theorem 1.2 in the case $\gamma \le 1$. $\square$

Let us now present the case $\gamma >1$, the strategy for which is similar to before. Fix $\epsilon >0$ and suppose that $\mu ^0\in \textsf{X}_{\alpha +\epsilon ,\gamma }^{\sigma _{0,r},\sigma _{0,q},r,\frac{2q}{q-1}}$ for $\sigma _{0,r},\sigma _{0,q}$ above the regularity thresholds $\kappa _{0,r},\kappa _{0,q}$ given by Proposition 4.1 and $1<q<\frac{d}{\gamma -1}$. Let us drop the subscript $\gamma$ from the notation $\textsf{X}_{\alpha +\epsilon ,\gamma }$, as $\gamma >1$ is fixed. In what follows, we assume that the parameters $d,\gamma ,r,q,s,\sigma _{0,r},\sigma _{0,q},\alpha ,\beta ,\nu$ satisfy all the constraints of Theorem 1.2. We also assume that

4.70 $$\begin{aligned} 0< \Vert \mu ^0\Vert _{\textsf{X}_{\alpha +\epsilon }^{\sigma _{0,r},\sigma _{0,q}, r,\frac{2q}{q-1}}} < \frac{\nu ^2-2\beta }{\max \{C_{mon,r},C_{mon,q}\}|{\mathbb {M}}|}, \end{aligned}$$

where $C_{mon,r}=C_r,C_{mon,q}=C_q>0$ are the constants from Proposition 4.1. Assuming a realization of *W* from $\Omega _{\alpha ,\beta ,\nu }$, Proposition 3.1 implies that given $r\ge 1$ sufficiently small depending on $d,s,\gamma$ and $0<\sigma <\frac{2s-1}{s}$ sufficiently large depending on $d,\gamma ,s,r$, there is a maximal solution $\mu$ to the Cauchy problem (3.1) with lifespan $[0,T_{\max ,\sigma ,\epsilon })$, such that $\mu$ belongs to $C_T^0\textsf{X}_{\phi +\epsilon }^{\sigma ,0,r,\frac{2q}{q-1}}$ for any $0\le T<T_{\max ,\sigma ,\epsilon }$. Here, we recycle the notation $T_{\max ,\epsilon ,\sigma }$ used above. Analogous to Lemma 4.5, we have the following relation for the maximal lifespans as we decrease $\epsilon$.

#### Lemma 4.6

Let $\mu$ be as above. There exists a constant $C>0$ depending on $d,\gamma ,r,q,s,\sigma ,\beta ,\nu$ such that for any $0\le \epsilon _2<\epsilon _1\le \epsilon$, the maximal times of existence $T_{\max ,\sigma ,\epsilon _1}, T_{\max ,\sigma ,\epsilon _2}$ of $\mu ^t$ as taking values in $\textsf{X}_{\phi ^t+\epsilon _1}^{\sigma ,0,r,\frac{2q}{q-1}}, \textsf{X}_{\phi ^t+\epsilon _2}^{\sigma ,0,r,\frac{2q}{q-1}}$, respectively, satisfy the inequality

4.71 $$\begin{aligned} T_{\max ,\sigma ,\epsilon _2} \ge T_{\max ,\sigma ,\epsilon _1} + C(|{\mathbb {M}}|\Vert \mu ^0\Vert _{\textsf{X}_{\alpha +\epsilon }^{\sigma _{0,r},\sigma _{0,q},r,\frac{2q}{q-1}}})^{-\frac{2s}{2s-\sigma s-1}}. \end{aligned}$$

#### Proof

Fix $0<\epsilon _2 <\epsilon _1\le \epsilon$. For any $\sigma _r \ge \sigma$ and $\sigma _q \ge 0$, it follows from Lemma 3.5 that $\mu$ is also a solution in $C_T^0\textsf{X}_{\phi +\epsilon _2}^{\sigma _r,\sigma _q,r,\frac{2q}{q-1}}$ for any $0\le T<T_{\max ,\sigma ,\epsilon _1}$. Furthermore, we have the quantitative bounds

4.72 $$\begin{aligned}&\Vert \mu ^t\Vert _{{\textsf{G}}_{\phi ^t+\epsilon _2}^{\sigma _r,r}} \le \frac{\lceil {\sigma _r-\sigma }\rceil !}{(\epsilon _1-\epsilon _2)^{\lceil {\sigma _r -\sigma }\rceil }} \Vert \mu ^t\Vert _{{\textsf{G}}_{\phi ^t+\epsilon _1}^{\sigma _r,r}} \qquad \forall \ 0\le t<T_{\max ,\sigma ,\epsilon _1}, \end{aligned}$$

4.73 $$\begin{aligned}&\quad \Vert \mu ^t\Vert _{{\textsf{G}}_{\phi ^t+\epsilon _2}^{\sigma _q,\frac{2q}{q-1}}} \le \frac{\lceil {\sigma _q}\rceil !}{(\epsilon _1-\epsilon _2)^{\lceil {\sigma _q}\rceil }} \Vert \mu ^t\Vert _{{\textsf{G}}_{\phi ^t+\epsilon _1}^{0,\frac{2q}{q-1}}} \qquad \forall \ 0\le t<T_{\max ,\sigma ,\epsilon _1}, \end{aligned}$$

which of course imply that $\Vert \mu \Vert _{C_T^0\textsf{X}_{\phi +\epsilon _2}^{\sigma _r,\sigma _q,r,\frac{2q}{q-1}}}$ is finite on any compact subinterval of $[0,T_{\max ,\sigma ,\epsilon _1})$. Choose $\sigma _r,\sigma _q$ such that

4.74 $$\begin{aligned} \sigma _{0,r} \ge \sigma _r> \kappa _{0,r} \quad \text {and} \quad \sigma _{0,q} \ge \sigma _q > \kappa _{0,q}, \end{aligned}$$

where $\kappa _{0,r},\kappa _{0,q}$ are the regularity thresholds of Proposition 4.1. Using the assumption (4.57), we can then apply Proposition 4.1 to conclude that the function $t\mapsto \Vert \mu ^t\Vert _{\textsf{X}_{\phi ^t+\epsilon _2}^{\sigma _r,\sigma _q,r,\frac{2q}{q-1}}}$ is strictly decreasing on the interval [0, *T*] for any $T<T_{\max ,\sigma ,\epsilon _1}$. In particular, since we can ensure $\sigma \le \sigma _r$ and $0\le \sigma _q$, it follows from this monotonicity and Lemma 3.5 that

4.75 $$\begin{aligned} \Vert \mu \Vert _{C_T^0\textsf{X}_{\phi +\epsilon _2}^{\sigma ,0,r,\frac{2q}{q-1}}} \le \Vert \mu \Vert _{C_T^0\textsf{X}_{\phi +\epsilon _2}^{\sigma _r,\sigma _q,r,\frac{2q}{q-1}}} \le \Vert \mu ^0\Vert _{\textsf{X}_{\alpha +\epsilon _2}^{\sigma _r,\sigma _q,r,\frac{2q}{q-1}}} \le \Vert \mu ^0\Vert _{\textsf{X}_{\alpha +\epsilon }^{\sigma _{0,r},\sigma _{0,q},r,\frac{2q}{q-1}}}\nonumber \\ \end{aligned}$$

for all $0\le T<T_{\max ,\sigma ,\epsilon _1}$. Let $C_{lwp,\sigma }$ be the constant from Proposition 3.1, and choose $T_* < T_{\max ,\sigma ,\epsilon _1}$ so that

4.76 $$\begin{aligned} T_{\max ,\sigma ,\epsilon _1}-T_* \le \frac{C_{lwp,\sigma }(|{\mathbb {M}}| \Vert \mu ^0\Vert _{\textsf{X}_{\alpha +\epsilon }^{\sigma _{0,r},\sigma _{0,q},r,\frac{2q}{q-1}}})^{-\frac{2s}{2s-\sigma s-1}}}{2}. \end{aligned}$$

Then by the same argument as in the proof of Lemma 4.5, we see that $\mu \in C_{T_0}^0\textsf{X}_{\phi +\epsilon _2}^{\sigma ,0,r,\frac{2q}{q-1}}$, where

4.77 $$\begin{aligned} T_0 \ge T_{\max ,\sigma ,\epsilon _1} + \frac{C_{lwp,\sigma }\left( |{\mathbb {M}}| \Vert \mu ^0\Vert _{\textsf{X}_{\alpha +\epsilon }^{\sigma _{0,r},\sigma _{0,q},r,\frac{2q}{q-1}}}\right) ^{-\frac{2s}{2s-\sigma s-1}}}{2}, \end{aligned}$$

which completes the proof of the lemma. $\square$

*Proof of Theorem* 1.2*for* $\gamma >1$. Fix $0<\epsilon '<\epsilon$. Let $\sigma ,\sigma _{0,r},\sigma _{0,q}$ be as above. If $T_{\max , \sigma , \epsilon '}<\infty$, then choosing $n\in {\mathbb {N}}$ such that

4.78 $$\begin{aligned} n C(|{\mathbb {M}}|\Vert \mu ^0\Vert _{\textsf{X}_{\alpha +\epsilon }^{\sigma _{0,r},\sigma _{0,q},r,\frac{2q}{q-1}}})^{-\frac{2s}{2s-\sigma s-1}} > T_{\max ,\sigma ,\epsilon '}-T_{\max ,\sigma ,\epsilon }, \end{aligned}$$

where *C* is the same constant as in the inequality (4.71), one can use the same argument from above, except now invoking Lemma 4.6, to derive a contradiction. Thus, $T_{\max ,\sigma ,\epsilon '}=\infty$.

We have shown that for any $0<\epsilon '<\epsilon$ and all $0<\sigma <\frac{2s-1}{s}$ sufficiently large depending on $d,s,\gamma ,r$, it holds that $\Vert \mu \Vert _{C_T^0\textsf{X}_{\phi +\epsilon '}^{\sigma ,0,r,\frac{2q}{q-1}}}<\infty$ for all $T>0$. By the arbitrariness of $\epsilon '$, Lemma 3.5 implies for any $T>0$, $\Vert \mu \Vert _{C_T^0\textsf{X}_{\phi +\epsilon '}^{\sigma _{0,r}+\frac{2}{r}, \sigma _{0,q}+\frac{q-1}{q},r,\frac{2q}{q-1}}} < \infty$. Using initial datum assumption (4.70), we can apply Proposition 4.1 on the interval [0, *T*] to obtain that the function $t\mapsto \Vert \mu ^t\Vert _{\textsf{X}_{\phi ^t+\epsilon '}^{\sigma _{0,r},\sigma _{0,q},r,\frac{2q}{q-1}}}$ is strictly decreasing on [0, *T*]. Since $T>0$ was arbitrary, we see that this monotonicity property holds on the entire interval $[0,\infty )$. Using the same monotone convergence argument from before, we complete the proof in the $\gamma >1$ case. $\square$

## Data Availability

Data sharing not applicable to this article as no datasets were generated or analysed during the current study.

## Footnotes

1 In the interests of completeness, we also mention that several works (e.g. [8, 63, 69]) have investigated the suppression of finite-time blow-up in the Patlak–Keller–Segel equation by *deterministic* perturbations of convective type.

2 This case is limited to dimensions $d\ge 2$, since there is no antisymmetric matrix (i.e. scalar) in dimension 1.

3 If $\mu ^0=0$, then by uniqueness, the solution $\mu$ is zero identically in time, hence strict monotonicity cannot hold in this trivial case.

4 If we work on ${\mathbb {T}}^d$ and replace $(1+|\nabla |^s)$ with $|\nabla |^s$, then mass is conserved.

5 Here, $\ell ^p$ denotes the space of sequences $x=(x_n)_{n=1}^\infty \in {\mathbb {R}}^{{\mathbb {N}}}$ such that $\Vert x\Vert _{\ell ^p}^p = \sum _{n=1}^\infty |x_n|^p < \infty$. It is elementary that for any $0<p_1\le p_2<\infty$, we have $\Vert x\Vert _{\ell ^{p_2}} \le \Vert x\Vert _{\ell ^{p_1}}$.
